# Alterations in TRN-anterodorsal thalamocortical circuits affect sleep architecture and homeostatic processes in oxidative stress vulnerable Gclm^−/−^ mice

**DOI:** 10.1038/s41380-022-01700-w

**Published:** 2022-07-28

**Authors:** Christina Czekus, Pascal Steullet, Albert Orero López, Ivan Bozic, Thomas Rusterholz, Mojtaba Bandarabadi, Kim Q. Do, Carolina Gutierrez Herrera

**Affiliations:** 1 Center for Experimental Neurology, Department of Neurology, Inselspital University Hospital, Bern, Switzerland; 2 Center for Psychiatric Neuroscience, Department of Psychiatry, Lausanne University Hospital, Site de Cery, CH-1008 Prilly-Lausanne, Switzerland; 3 Department for Biomedical Research, University of Bern, Bern, Switzerland; 4 Present Address: Department of Biomedical Sciences, University of Lausanne, Lausanne, Switzerland

**Keywords:** Neuroscience, Schizophrenia

## Abstract

Schizophrenia is associated with alterations of sensory integration, cognitive processing and both sleep architecture and sleep oscillations in mouse models and human subjects, possibly through changes in thalamocortical dynamics. Oxidative stress (OxS) damage, including inflammation and the impairment of fast-spiking gamma-aminobutyric acid neurons have been hypothesized as a potential mechanism responsible for the onset and development of schizophrenia. Yet, the link between OxS and perturbation of thalamocortical dynamics and sleep remains unclear. Here, we sought to investigate the effects of OxS on sleep regulation by characterizing the dynamics of thalamocortical networks across sleep-wake states in a mouse model with a genetic deletion of the modifier subunit of glutamate-cysteine ligase (*Gclm knockout*, KO) using high-density electrophysiology in freely-moving mice. We found that *Gcml* KO mice exhibited a fragmented sleep architecture and impaired sleep homeostasis responses as revealed by the increased NREM sleep latencies, decreased slow-wave activities and spindle rate after sleep deprivation. These changes were associated with altered bursting activity and firing dynamics of neurons from the thalamic reticularis nucleus, anterior cingulate and anterodorsal thalamus. Administration of N-acetylcysteine (NAC), a clinically relevant antioxidant, rescued the sleep fragmentation and spindle rate through a renormalization of local neuronal dynamics in *Gclm* KO mice. Collectively, these findings provide novel evidence for a link between OxS and the deficits of frontal TC network dynamics as a possible mechanism underlying sleep abnormalities and impaired homeostatic responses observed in schizophrenia.

## Introduction

Schizophrenia (SZ) is a mental disorder that dramatically alters the life quality of the individuals and their relatives on a daily basis. Early diagnosis includes the presence of positive (i.e., hallucinations, delusions) and negative (i.e., apathy, anhedonia, social withdrawal) symptoms in addition to deficits in cognition, sensory and emotional processing. In addition, sleep disturbances are commonly observed in subjects with SZ and associated with exacerbated psychotic symptoms and poor clinical outcomes [1–5]. Interestingly, they are often reported during the prodromal phase preceding a first-episode of psychosis [5–8]. Sleep perturbations commonly include increased sleep onset latency, reduced duration of non-rapid eye movement (NREM) sleep stages 1 and 3, and fragmentation of the sleep-wake cycle architecture, while alterations of rapid eye movement (REM) sleep have been less consistently reported [3, 6–10]. Polysomnography studies revealed a reduction of slow waves (SWs) and spindles during NREM sleep in subjects with SZ [1, 2, 9–18] or high-risk of developing psychosis [2, 11, 19]. Changes in the slow-wave activity (SWA) during NREM sleep suggested a lack of integrity of homeostatic regulatory mechanisms in SZ, as revealed by impaired homeostatic regulation of sleep after sleep deprivation [3, 6, 20].

Abnormal sleep spindles and SWs often reflect aberrant coordinated activity within thalamo-cortical (TC) networks and are associated with the neuropathophysiology of SZ [12, 21–23]. The interplay of thalamic and cortical networks modulates local topography of sleep oscillations including SWA and spindles [24–29]. During NREM sleep, high amplitude SWA nests higher frequency oscillations in humans, cats, and rodents that result from a precise orchestration of neuronal dynamics between inhibitory and excitatory neurons within thalamocortical networks [30] (for review [31–35]). The accumulation of sleep pressure during wakefulness, or fragmented sleep, dissipates during sleep via a phenomenon called sleep homeostasis that involves the upregulation of SWA [36–42]. SWA are more prominent in frontal cortices and have been linked to cognitive processing including memory consolidation in human [9, 39, 43].

Spindles emerge from thalamocortical interactions as transient and distinct oscillations (9–16 Hz) and reflect sleep stability in both rodents and humans [24, 33, 44–48]. In rodents, they are commonly defined as waxing and waning oscillations of variable peak amplitude (~100 µV) and duration (>400 ms) that often coincide with the UP-state of cortical SWs, or after a K-complex [49, 50]. Spindles are generated by the transient burst firing of gamma-aminobutyric acidergic (GABAergic) neurons (mostly parvalbumin (PV) expressing neurons) in the thalamic reticular nucleus (TRN) [51, 52] that subsequently evokes bursting activity in TC relay cells and generates typical spindling activity within the TC networks [6, 8, 11, 13, 24, 53–55]. Burst firing in TRN neurons is triggered by the activation of T-type calcium channels – namely Cav3.3 [56]. Both intrinsic properties of TRN cells [13] and functional inputs they receive from cortical origins are sufficient to generate spindle-like activity, whereas cortical inputs to the TRN cells and number of action potentials per burst in TRN cells influence spindle length [57, 58]. Functionally, a correlative role for spindles in memory consolidation, intelligence, and cognition has been proposed [1, 46, 48, 49, 59–64], suggesting that the integrity of spindles is essential to higher brain functions.

Importantly, the cellular and molecular mechanisms underlying sleep disturbances and altered brain oscillations during NREM sleep in SZ remain unclear. Accumulating evidence indicates that redox dysregulation and susceptibility to oxidative stress (OxS)—i.e., altered antioxidant systems, reduced antioxidant capacities and increased levels of OxS markers - are among the pathological mechanisms that contribute to the emergence of psychosis [65–68]. Thus, OxS alters the function of redox-sensitive proteins including NMDA receptors and T-type calcium channels, impairs PV interneuron activity and promotes inflammation processes, all of which represent hallmarks of the molecular and cellular pathological mechanisms associated with development of SZ. Moreover, OxS preferential impacts inhibitory PV neurons including those located TRN and anterior cingulate cortex (ACC), key contributors to the TC oscillations, sleep and sleep homeostasis in both rodents and humans [65, 69, 52]. Indeed, energy metabolism and redox-dependent processes are intrinsically linked to the circadian cycle, sleep perturbations [70, 71], and maintenance of proper homeostasis [72–74]. The implication of redox dysregulation in the pathology of SZ is further supported by the beneficial effects of the antioxidant and glutathione (GSH) precursor, N-acetylcysteine (NAC), on negative and positive symptoms, as well as cognition in patients [75].

The goal of this study was to test whether redox dysregulation favors sleep instability and disrupts sleep homeostasis, including SWA and spindles, through altered local neuronal activity in TC circuits during NREM sleep. To assess this, we characterized the sleep architecture and TC neuronal dynamics and oscillations in a mouse model carrying a genetic deletion of the modifier subunit of glutamate-cysteine ligase (*Gclm knockout*, KO) using multi-site in-vivo electrophysiology in freely-moving *Gclm* mice. These mice have a functional deletion of the modulatory subunit of the key GSH synthesizing enzyme resulting in low brain GSH levels and decreased number of PV-immunoreactive neurons in TRN and ACC [65]. Importantly, TRN neurons from *Gclm* KO mice are less prone to burst firing activity in ex-vivo brain slices [76], due to an OxS-induced decrease of T-type calcium currents [77]. We found that *Gclm* KO mice displayed a fragmented sleep architecture and impaired local sleep homeostasis in non-sensory TC networks in baseline and after sleep deprivation that were largely rescued by a clinically relevant NAC treatment.

## Methods

### Animals

We used male *Gclm*^*−/−*^ (KO) and *Gclm*^*+/+*^ (wild-type, WT) mouse littermates from a breeding maintained at the “Center d’Etude du comportement” at Lausanne University Hospital, originally reported in [78]. Animals were housed in individual custom-designed polycarbonate cages at constant temperature (22 ± 1 °C), humidity (40–60%) and circadian cycle (12-h light-dark cycle, lights on at 08:00). Food and water were available ad libitum. Animals were treated according to protocols and guidelines approved by the Veterinary office of the Canton of Bern, Switzerland (License number BE 49/17 and BE 18/2020). Criteria for inclusion and exclusion are included in supplementary materials and methods.

### Instrumentation

Animals of 10–16 week old were instrumented with two EEG electrodes (frontal: AP − 2.0 mm, ML + 2.0 mm, and parietal: AP − 3.0 mm, ML + 2.7 mm) together with tetrode wires inserted into ACC (AP + 1.2 mm, ML + 0.2 mm, DV − 1.5 mm), anterior dorsal thalamus (AD) (AP − 0.85 mm, ML + 0.75 mm, DV − 2.75 mm), anterior TRN (AP − 0.8 mm, ML + 1.7 mm, DV − 3.5 mm), ventral posterolateral nucleus (VPL) (AP − 1.6 mm, ML − 1.82 mm, DV − 3.6 mm), and sensory cortex (Brr) (AP − 1.8 mm, ML + 2.8 mm, DV − 1.5 mm). Two bare-ended wires were sutured to the trapezius muscle on each side of the neck to measure electromyography (EMG) signals. Details on the surgical procedures and materials are included in supplementary materials and methods.

### In vivo electrophysiological recordings

For all multisite recordings, mice were connected to a tethered digitizing headstage (RHD2132, Intan Technologies) and data was sampled at 20 kHz and recorded with open source software (RHD2000 evaluation software, Intan Technologies). For details see supplementary materials and methods.

### Histological characterization and immunohistochemistry

Confirmation of electrode placement (Fig. 2 and Supplementary Figs. 2, 5) was carried out as described in supplementary materials and methods. Immunolabeling of fast-spiking PV GABAergic interneurons in the ACC and TRN were performed as described in supplementary materials and methods.

### Determination of vigilance state

We scored vigilance states manually, blind to the experimental conditions, in 1 s epochs using the concurrent evaluation of EEG and EMG signals and power band analysis. Vigilance states have been defined as previously described and are detailed in supplementary materials and methods. Data analyses were carried out using custom scripts written in MATLAB^®^ (R2018b, MathWorks, Natick, MA, USA). Furthermore, built-in functions from Wavelet and Signal Processing toolboxes of MATLAB were used.

### Spectral analysis

Power spectral density (PSD) to estimate delta power was carried it out with the Welch’s method (pwelch, MATLAB R2018b Signal Processing Toolbox) using 8 s windows having 75% overlap. Value of total power across frequencies was used for normalization between animals. A detailed description of the power calculations for slow-wave bands, spindle detection, cross-correlation and phase frequency coupling and modulation indexes are described in details in supplementary material and methods.

### Single unit analysis

Multiunit activity from tetrodes with confirmed resistance between 100–200 KΩ at the time of recording was first extracted from the signal bandpass-filtered at 600–4000 Hz using a fourth-order elliptic filter and 0.1 dB passband ripple with a − 40 dB stopband attenuation. Filtering, detection threshold and clustering were performed as previously described [26]. For details see supplementary material and methods.

### NAC treatment

In a separate cohort of *Gclm* KO mice, NAC (Fluimucil, Zambon, Switzerland) was provided in the drinking water at 2.4 g/L from few days prior to the electrode implantation (at postnatal week 8) till the end of the experiment. Fresh NAC solutions were renewed every other day [79, 80].

### Statistical methods

A detailed description of the statistical analyses is provided in supplementary materials and methods. Note that experimenter was blinded to the genotype of the animals.

## Results

### Altered sleep regulation in *Gclm* KO mice

**Fig. 1:**
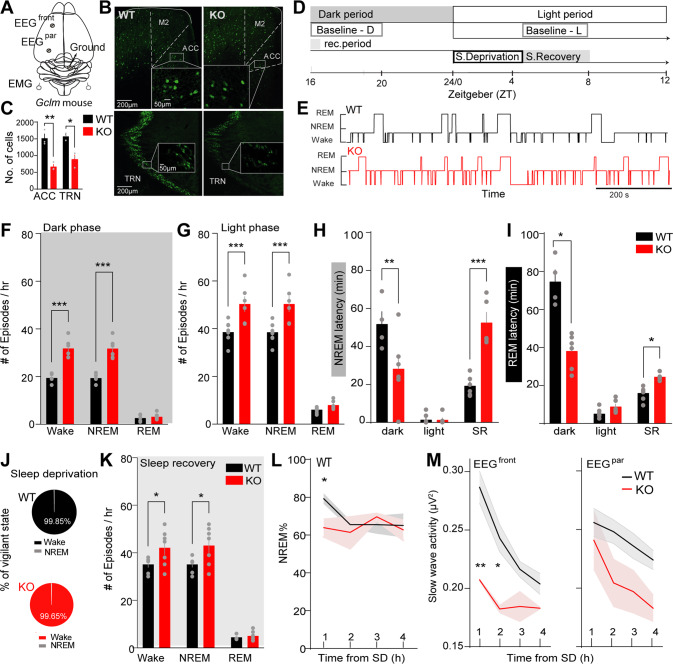
Characterization of Sleep and Sleep homeostatic responses in *Gclm* WT and KO mice. **A** Schematic representation of electroencephalogram (EEG) electrode placement. **B** Micrographs of hemi-sections of brain slices showing immune-reactive labeled parvalbumin (PV + ) neurons in the ACC (top panels) and TRN (bottom panel) of adult *Gclm*^*+/+*^ (WT) and *Gclm*^*−/−*^ (KO) mice. Dotted square lines delimits the magnified area. **C** Significant differences in number of PV + cells in WT (*n* = 3) and KO (*n* = 3) mice quantified in the TRN (*P = 0.0166) and ACC (**P = 0.0077). **D** Experimental time line. EEG/EMG signals were recorded for 4 hs in the light (Baseline, BL) and dark period from Zeitgeber time (ZT) 4–8 and 16–20, respectively, to represent wake/sleep cycles. A protocol of 4 hs of sleep deprivation (SD) (ZT 4–8) and the 4 h of the sleep recovery period (SR) (ZT 4-8) were recorded. **E** Representative hypnograms of the sleep architecture are shown for WT (black trace) and *Gcml* KO (red trace) mice. **F**, **G** Number of episodes per h of each vigilance state during the dark period (**F**) (Wake and NREM sleep; ***P < 0.001, *t* = 7.3 DF = 27, *n* = 5 WT, 6 KO), and the light period (**G**) (Wake and NREM sleep; ***P < 0.001, *t* = 7.3 DF 27, *n* = 8 WT, 7KO). **H** Latencies to the first consolidated NREM sleep episode for WT (black) and KO (red) during the dark, light and SR periods (SR **P = 0.007, *t* = 5.5 DF = 5.16, *n* = 6, 5 and for dark and SR, *n* = 8 WT, 6 KO for light). **I** Latencies to the first REM sleep episode during the light, dark and SR phases (Dark: *P = 0.02 *t* = 6.02 DF = 3, *n* = 4, 6; light: *n* = 8 WT, 6 KO; SR: *P = 0.037, *t* = 4.33 DF 4, *n* = 8, 5 for WT and KO, respectively). **J** Circular graph representation of average of the total time (percentage) of Wake (denoted on the graph) and NREM sleep during the 4 h of sleep deprivation in the WT (black) and in *Gcml* KO (red). No REM sleep was scored during the SD. **K** Number of episodes per hour of each vigilance state during the first hour of SR (Wake: *P = 0.04, *t* = 3.75 DF 72 and NREM sleep: *P = 0.01, *t* = 4.2 DF = 72; *n* = 7, 7 for WT and KO, respectively). **L** Percentage time of NREM sleep during each of the first 4 hs of SR. starting from the first stable NREM sleep (>20 s) (*P = 0.02, *t* = 2.95 DF 25, *n* = 5 WT, 5 KO). **M** Normalized time progression of slow-wave activity (SWA) during the first 4 hs SD showing significant difference between genotypes on the frontal EEG (EEG^front^) (left, **P = 0.009 *t* = 5.7 DF = 5.049, and *P = 0.039 *t* = 4.9 DF = 5.7), but not in the parietal EEG (EEG^par^) electrodes (right) (*n* = 6 WT, 4 KO). All graphs display the averaged of the mean values per animal + /− s.em; multiple comparisons were carried out using two-way ANOVA and Bonferroni’s multiple comparison test.

Consistent with previous studies, we found that *Gclm* KO mice displayed decreased number of PV immunoreactivity in ACC and TRN (Fig. 1A–C, and as in [76, 77]). Using longitudinal polysomnographic recordings across the dark-light cycle (baseline condition, BL), we observed a higher number of wake and NREM sleep episodes during both dark and light period in *Gclm* KO mice as compared to controls (Fig. 1F, G) and reduced latencies to REM sleep in the dark phase (Fig. 1H,I). Concomitant to the increased number of episodes, the mean duration of Wake and NREM sleep episodes were significantly reduced in *Gclm* KO mice during the dark and light periods respectively (Supplementary Fig. 1A). However, the total amounts of each vigilance state remained similar in both genotypes (Supplementary Fig. 1B). Together, these data revealed a severe sleep fragmentation in *Gclm* KO mice accompanied by a decreased duration of NREM sleep episodes.

Sleep fragmentation triggers the homeostatic response that includes a decrease in the time to fall asleep and an increase in sleep duration and sleep depth in mice [81]. To test this, we characterized the sleep homeostasis of *Gclm* KO and control animals using a 4-h gentle handling sleep deprivation (SD) procedure starting at light onset [81–83], and recorded the subsequent sleep recovery (also called sleep rebound, SR; Fig. 1D). During the sleep deprivation, all mice remained awake for >99% of the time, irrespective of the genotype (Fig. 1J and Supplementary Fig. 1C). Interestingly, during the subsequent SR, the number of Wake and NREM episodes per hour was significantly higher (Fig. 1K), while the latency to NREM and REM sleep during SR were significantly longer in *Gclm* KO mice as compared to controls (Fig. 1H,I). A time-course analysis further revealed a lack of homeostatic changes on the total time spent in NREM (Fig. 1L), REM sleep and Wake (Supplementary Fig. 1D) during the 4 h following SD in *Gclm* KO mice as compared to controls. Consistent with sleep recovery described in rodents and human [24, 26, 36, 42, 84–86], *Gclm* KO mice failed to express an upregulation of SWA during the first hour of SR as compared to controls. The difference of SWA between both genotypes was prominent in frontal but *not* parietal regions (Fig. 1M). Collectively, these results suggested a lack of homeostatic response in *Gclm* KO mice, possibly via a dysregulation of the thalamic and cortical networks underlying the modulation of sleep SWA.

### Deficits in homeostatic regulation of NREM sleep delta and spindles in *Gclm* KO mice

**Fig. 2:**
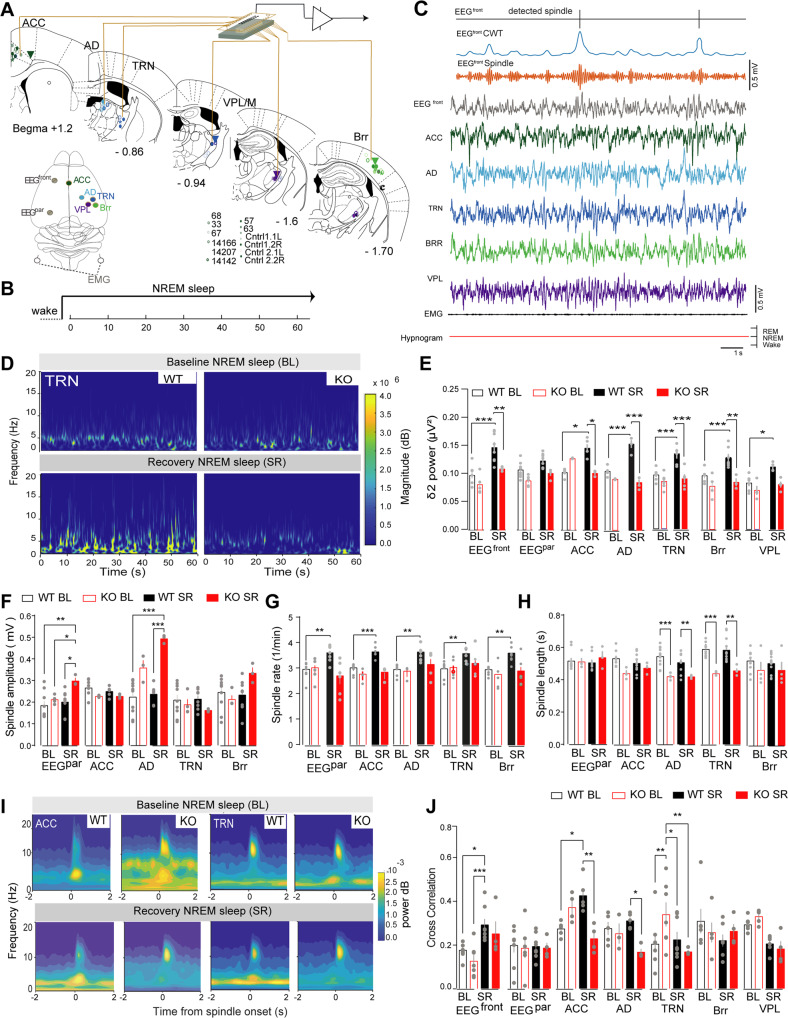
Topographical characteristics analysis of NREM sleep SWA and Spindles in *Gclm* WT and KO mice during BL and SR periods. **A** *Right top*, schematic representation of the placement of EEG and EMG electrodes, and tetrodes in thalamocortical (TC) networks for multisite recordings in freely-behaving mice: anterior cingulate cortex (ACC), anterio-dorsal thalamus (AD), ventral posterolateral nucleus of the thalamus (VPL), thalamic reticularis nucleus (TRN) and somatosensory cortex (Brr). *Left*, schematic anterior posterior representation of coronal sections taken from *Paxinos and Franklin* (G Paxinos and KBJ Franklin, *Paxinos and Franklin’s the Mouse Brain in Stereotaxic Coordinates*, 2019.) atlas summarizing the final position of the electrodes in all animals included in the analyses. **B** Experimental timeline for the NREM sleep analysis of the power spectra shown in *D*. **C** Representative traces of detected spindles (top), normalized continuous wavelet transform (CVT) energy using the complex Morlet and frequency B-Spline functions (blue), filtered EEG signals at spindle range (10–16 Hz, orange) and unfiltered EEG (gray), LFP traces from ACC (green), AD (light blue), TRN (blue), Brr (light green), VPL (purple) EMG (black), and hypnogram (red). **D** Representative time-frequency of local power spectra recorded from EEG electrodes and tetrodes within the TC networks in *WT* and *KO* animals during baseline (BL) (right) and sleep recovery (SR) (left). **E** Normalized Delta 2 (δ2) power (2.5–3.5 Hz) during NREM sleep BL and the first hour of SR taken from the EEG and tetrodes within the TC networks: Significance between genotypes: ***P < 0.001, F = 48.92 DF *n* = 3 DFd = 25; effect between sleep conditions (BL- SR) ***P < 0.001, F = 4.03 Dfn 18, DFd 135; EEG^front^ [WT BL] - [WT SR] **P = 0.004, [WT SR] - [KO SR] *P = 0.015; EEG^Par^ [WT SR] - [KO SR] *P = 0.012; ACC [WT BL] - [KO BL] ***P < 0.001, [WT BL] - [WT SR] ***P < 0.001, [WT SR] - [KO SR] **P = 001; AD [WT BL] - [WT SR] ***P < 0.001, [WT SR] - [KO SR] **P < 0.001; TRN [WT BL] - [WT SR] **P = 0.002, [WT SR] - [KO SR] **P = 0.002; Brr [WT BL] - [WT SR]^]^ **P = 0.005, [WT SR] - [KO SR] **P = 0.001; VPL [WT BL] - [WT SR] ***P < 0.001, [WT SR] - [KO SR] **P = 0.004. **F** Spindle amplitude during BL and SR NREM sleep: (effect ***P = < 0.001, F = 10.59 DFn = 6, DFd = 137), EEG^front^: [WT BL] - [KO SR] **P = 0.006, [KO BL] - [KO SR] *P = 0.03, [WT SR] - [KO SR] *P = 0.015; AD: [WT BL] - [KO BL] **P = 0.006, [WT BL] - [KO SR] ***P, 0.001, [KO BL] - [WT SR] *P = 0.03, [KO BL] - [KO SR] *P = 0.04, [WT SR] - [KO SR] ***P, 0.001. **G** Spindle rate during BL and SR NREM sleep [WT BL] - [WT SR] in EEG^Par^: **P = 0.007; ACC: ***P < 0.001; AD: **P = 0.003; TRN: **P = 0.007; Brr: **P = 0.001. Effect between BL and SR ***P < 0.001, F = 18.01 DFn = 3 DFd = 24. **H** Spindle length during BL and SR NREM sleep: (effect ***P = < 0.001, F = 18.22 DFn = 3, DFd = 24), EEG^front^: [WT BL] - [KO BL] **P = 0.003, [WT SR] - [KO SR] ***P > 0.001; AD: [WT BL] - [KO BL] **P = 0.003, [WT SR] - [KO SR] **P = 0.007; TRN: [WT BL] - [KO BL] ***P < 0.001, [WT SR] - [KO SR] ***P > 0.001. Numbers for *F- G*, EEGs: BL *n* = 8, 6 and SR *n* = 8, 5; ACC: BL *n* = 8, 6 and SR *n* = 8, 6; AD: BL *n* = 6, 5 and SR *n* = 8, 4; TRN: BL *n* = 8, 6 and SR *n* = 8, 6; Brr: BL *n* = 8, 6 and SR *n* = 8, 6; and VPL: BL *n* = 8, 6 and SR *n* = 8, 6 for WT and KO mice, respectively. **I** Representative spectrogram of time-frequency coherence between SWs (delta, 0.5–4 Hz) and spindle envelops in the ACC and TRN during BL and SR NREM sleep. **J** Normalized cross-correlation between SWs and spindles during BL and SR in all recorded locations: [WT BL] - [WT SR] *P = 0.015, [KO BL] - [WT SR] ***P < 0.00; ACC: [WT BL] - [WT SR] *P = 0.010, [WT SR] - [KO SR] ***P < 0.001; AD: [WT SR] - [KO SR] *P = 0.032; TRN: [KO BL] - [KO SR] *P = 0.036, [WT SR] - [KO SR] *P = 0.047. Animals numbers: EEG: BL *n* = 8, 7 and SR *n* = 8, 4; ACC: BL *n* = 5, 4 and SR *n* = 4, 5; AD: BL *n* = 5, 3 and SR *n* = 5, 4, TRN: BL *n* = 7, 7 and SR *n* = 7, 5; Brr: BL *n* = 6, 5 and SR *n* = 7, 4; and VPL: BL *n* = 6, 4 and SR *n* = 6, 4 for WT and KO mice, respectively. All data are represented by the mean ± s.e.m. Multiple comparisons differences were calculated using two-way ANOVA with Bonferroni’s multiple comparisons test.

To further investigate the alterations of NREM sleep regulation in *Gclm* KO mice, we compared the SWA and spindle features during NREM sleep across both spontaneous (baseline, BL) sleep and SR. A multisite electrophysiological approach was used to study local oscillations, neuronal activity and network interactions. [36] Cortical EEG signals were recorded concurrently with depth local field potentials (LFP) in fronto-thalamic circuits (ACC and AD) and TRN, and sensory TC circuits (VPL and Brr) during consolidated NREM sleep episodes of freely-moving mice (Fig. 2A–C, Supplementary Fig. 2A). Delta oscillations, that represent a predominant part of SWA, were split into δ1 (0.75–1.75 Hz) and δ2 (2.75–3.5 Hz). While δ1 is relatively insensitive to sleep homeostasis, the amplitude of δ2 directly depends on the time spent awake and correlates with activity in the mediodorsal thalamus and prefrontal cortex (PfC) [36]. During BL sleep, we found no significant differences of δ2 amplitude between experimental groups (Fig. 2D,E). As previously reported [36], δ2 amplitude was significantly increased in frontal cortices and locally in thalamocortical networks of WT mice during the SR period. In contrast, *Gclm* KO littermates showed no upregulation of δ2 amplitude as compared to controls (Fig. 2E). As expected, the power of δ1 was higher during SR as compared to BL in cortical regions of both WT and KO mice with no significant difference between genotypes. However, *Gclm* KO mice displayed significantly higher δ1 in TRN during BL sleep and in somatosensory TC networks during SR when compared to WT mice (Supplementary Fig. 2B). No significant differences were found for δ1 and δ2 power during the dark phase across recording sites in both WT and KOs (Supplementary Fig. 2C).

We then investigated how spindles were affected in spontaneous NREM and NREM sleep during SR in *Gclm* mice using an automated detection spindle method [24] (Fig. 2C and Supplementary Fig. 3A). We found no significant differences in amplitude of spindles between genotypes during BL. In contrast, during the SR period, spindle amplitude in the parietal EEG and AD electrodes was increased in *Gclm* KO mice as compared to WT mice (Fig. 2F and Supplementary Fig. 3B) [24]. Interestingly, we observed that spindle rates recorded from both cortical EEG and local field potentials (LFPs) within TC networks during spontaneous NREM sleep and NREM to REM sleep transitions were similar between WT and KO animals (Fig. 2G, Supplementary Fig. 3C, E). However, in contrast to the increase of spindle rate during SR in WT mice [24], *Gclm* KO mice lacked a proper homeostatic upregulation of spindle rate during SR (Fig. 2G and Supplementary Fig. 3C). Of note, spindle length was significantly shorter in the frontal EEG, AD and TRN in *Gclm* KO as compared to WT mice during both BL and SR sleep (Fig. 2H and Supplementary Fig. 3D).

Differences in SWA amplitude and spindle dynamics in *Gclm* KO mice were suggestive of possible alterations in the interactions between these two types of oscillatory activity. To assess the temporal relationship between SWA and spindles that exists locally in the TC circuits during NREM sleep, we extracted SWA (0.5–4 Hz) and spindle envelopes (Fig. 2I, Supplementary Fig. 3F) and computed a normalized cross-correlation between the peak of the SW and the spindle envelopes within each recorded brain region (see supplementary materials and methods). During BL sleep, CFCs were not significantly different between genotypes (Fig. 2J). Moreover, consistent with previous reports [24], we observed an increase of cross-correlation index in the ACC in WT mice during SR whereas an opposite modulation was shown by KO mice in ACC as well as in AD and TRN (Fig. 2J). Collectively, these results suggested that the organization of neuronal synchrony within local circuits in frontal cortex and AD, but not sensory TC network, is altered in *Gclm* KO as compared to WT mice during SR.

**Fig. 3:**
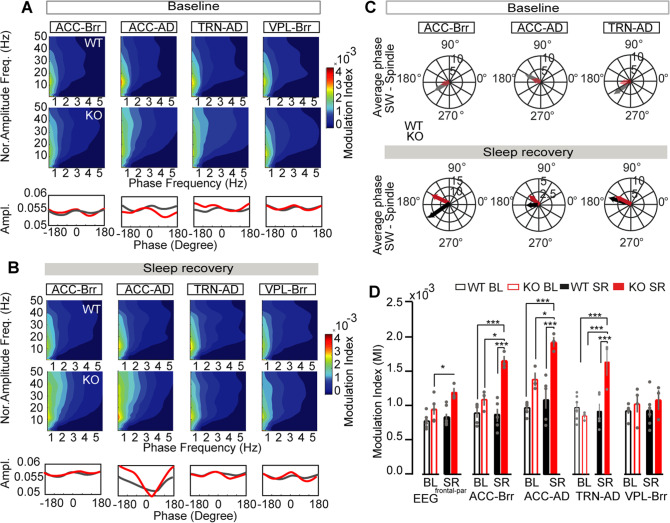
Topographic analysis of brain dynamics in *Gclm* WT and KO mice. **A**, **B**. Phase-amplitude coupling between delta (0.5–4 Hz) and spindle oscillations (10–16 Hz) along the following connected TC areas (ACC-AD, ACC-Brr, TRN-AD, VPL-Brr) during (**A**) BL NREM sleep and (**B**) the first h of SR. At the top are averaged phase-amplitude comodulograms (mean normalized amplitude distribution over phase bins) computed for the connected TC areas. At the bottom are normalized phase degree histograms of WT (gray) and KO (red) fitted curves. Numbers: *n* = 8 WT, 7 KO. **C** Circular representation of average phase distribution of SW (first pair)-spindle (second pair) peak coupling between two brain areas (pairs) in WT (black line) and KO (red line) during baseline (top panel) and detected event during the NREM of the SR (bottom panel). Note that peak of SW and spindles was taken at 180 degrees. See Supplementary Fig. 3F for phase distributions. **D** Average modulation index (MI) during BL NREM sleep and the first h SR computed for EEG front calculated statistical differences show an effect between genotypes ***P < 0.001, F = 28.77 DFn = 5, DFd= 86 and between thalamic nuclei as follows: EEG^front^ - EEG parietal (^par^) signals, ACC - Brr, ACC - AD, TRN - AD, and VPL - Brr LFPs (WT: EEG front-par: [WT BL] - [KO SR] *P = 0.016; ACC-Brr: [WT BL] - [KO SR] ***P < 0.001; [WT SR] - [KO SR] ***P < 0.001, [KO BL] - [KO SR] *P = 0.013; ACC-AD: [WT BL] - [KO SR] ***P < 0.001; [WT SR] - [KO SR] ***P < 0.001, [KO BL] - [KO SR] *P = 0.020; TRN-AC: [WT BL] - [KO SR] ***P < 0.001; [WT SR] - [KO SR] ***P < 0.001, [KO BL] - [KO SR] ***P < 0.001, *n* = 7, 5, 5, 6, 6 and *n* = 5, 3, 3, 3, 4 for WT and KO, respectively). Multiple comparisons differences were calculated using two-way ANOVA with Bonferroni’s multiple comparisons test. All results are represented by the mean + /− s.e.m.

Next, we investigated the putative long-range modulation of distant connected brain areas. Based on previous findings on TC network connectivity [24, 26, 87], a modulation index was used to measure Phase-Amplitude Coupling (PAC) for the following pairs of regions: intra-cortical (EEG^front^ - EEG^par^; ACC - Brr), intra-thalamic (TRN - AD), high-order (ACC - AD) and sensory TC (VPL - Brr) (Fig. 3). While no differences were found in BL, during the sleep recovery period we observed a significantly higher modulation index at low frequencies (0.1–2.5) and increased in phase amplitude in ACC - AD (Fig. 3B). Then, we computed the averaged phase coupling between SWs (first pair) and spindle amplitude (second pair) in another distant area. This analysis revealed an atypical phase coupling in ACC - Brr SR (Fig. 3C). Finally, modulation index of the different selected pairs showed stronger PAC between ACC - Brr, ACC -AD and TRN - AD in *Gclm* KO as compared to WT mice but not in other pairs (Fig. 3D and Supplementary Fig. 3G). These data further confirmed the specific alterations of TC network dynamics in *Gclm* KO mice during SR.

### Altered TC neuronal spike dynamics in *Gclm* KO mice

**Fig. 4:**
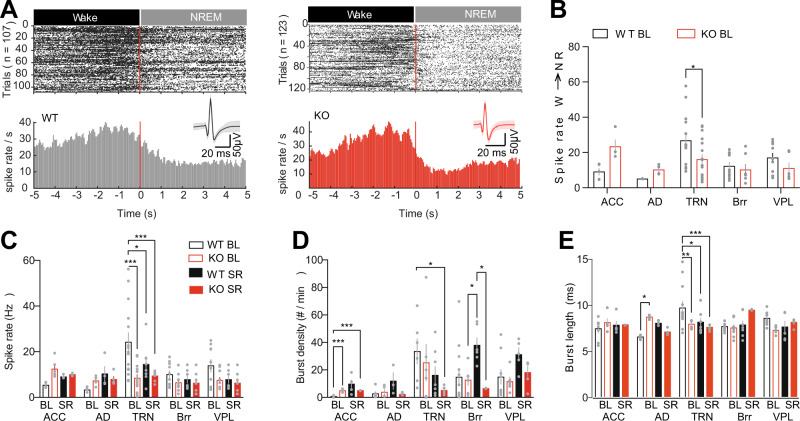
Spike activity within thalamocortical networks in *Gclm* WT and KO mice. **A** Raster plot of the spiking activity during Wake to NREM transitions isolated from tetrodes implanted in the TRN of WT (107 transitions) and KO (123 transitions). Bottom, histogram of spiking rate per sec during the transition for a representative isolated unit from WT (black) and KO mouse (red), respectively. **B** Averaged spike rate during BL NREM sleep and the first h of SR in TC circuits: ACC (*n* = 5, 4), AD (*n* = 2, 4), TRN (*P = 0.016, t = 3.04, DF = 70, *n* = 15, 17), Brr (*n* = 10, 7) and VPL (*n* = 10, 6) of WT (empty black graph bars) and KO (empty red graph bars) animals respectively. **C** Average spike rate during BL NREM sleep and the first h of SR in TC circuits: ACC (BL: *n* = 5, 6 and SR *n* = 4, 3); AD (BL: *n* = 5, 4 and SR *n* = 4, 5); TRN ([WT BL] - [KO BL] ***P < 0.001 t = 0.86 DF = 9.13, [WT BL] - [WT SR] *P = 0.011 t = 0.78, [WT BL] - [WT SR] ***P < 0.001 t = 0.91 DF = 130, BL: *n* = 15, 14 and SR *n* = 8, 9), Brr (BL: *n* = 10, 10 and SR *n* = 7, 7); VPL [WT BL] - [KO SR] *P = 0.036 (BL: *n* = 10, 10 and SR *n* = 7, 7) for WT and KO mice, respectively. **D** Averaged burst density from isolated during BL NREM sleep and the first h of SR in TC circuits: ACC: [WT BL] - [KO BL] **P < 0.001 t = 8.85 DF = 6.19, [WT BL] - [KO SR] **P < 0.001 t = 18.58 DF = 8.08; TRN: [WT BL] - [KO SR] **P = 0.0012; Brr [KO BL] - [WT SR] *P = 0.026, t = 3.75 DF = 9.24, [WT SR] - [WT SR] *P = 0.013 t = 5.82, DF 5.03 for WT and KO mice, respectively. **E** Summary data of the burst length taken from AD: [WT BL] - [KO BL] *P = 0.028, t = 2.84, TRN: [WT BL]- [KO BL] **P = 0.008, t = 2.94, [WT BL] - [WT SR] *P = 0.024, [WT BL] - [KO SR] **P = 0.003, t = 3.58, DF = 101 for WT and KO mice, respectively. Significance levels were calculated using two-way ANOVA and Bonferroni’s multiple comparison test. All data are represented by the mean + /− s.e.m.

As TRN neurons of *Gclm* KO mice displayed reduced bursting and decreased T-type calcium currents in ex vivo recordings (see [76, 77] and accompanying manuscript), we characterized the spiking activity of thalamic and cortical neurons across sleep-wake states and their transitions in Gclm KO and control mice (Fig. 4 and Supplementary Fig. 4). Spiking activity of TRN neurons was significantly lower during the Wake to NREM sleep transitions (Fig. 4A, B) while neuronal spiking was higher in the ACC and AD during NREM- REM sleep transitions (Supplementary Fig. 4). By contrast, no significant changes were found in the Brr or VPL. Interestingly, spiking activity of TRN cells was also lower during wakefulness and REM sleep in the TRN and higher in AD and ACC of KO as compared to control mice (Supplementary Fig. 4 and Supplementary Table 1). During NREM sleep, TRN single-unit activity, burst density and burst length were decreased in BL and SR (Fig. 4C–E) similar to observations from ex-vivo preparation [77].

### Pharmacological rescue of sleep and sleep spindles in *Gclm* KO mice

**Fig. 5:**
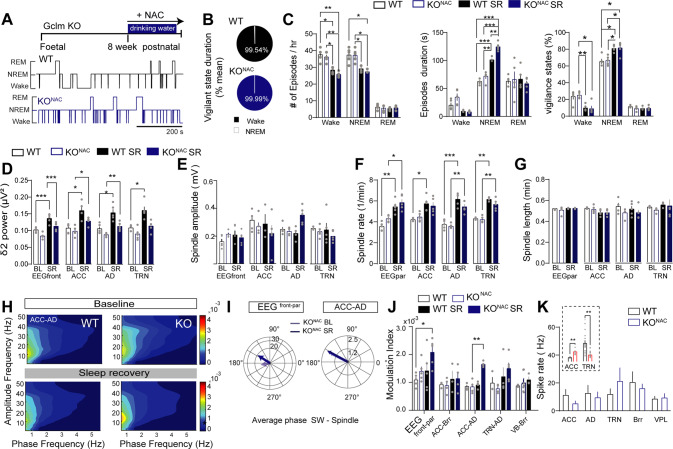
Sleep and NREM oscillatory dynamics after NAC treatment. **A** Top: experimental timeline of N-Acetylcysteine (NAC) treatment in *Gclm* KO (KO^NAC^) animals. Bottom: hypnogram of WT (black) and KO^NAC^ (blue) describing sleep architecture over a period of 10 min during the light period (BL). **B** Quantification of vigilance states during SD for WT (black) and in KO ^NAC^ (blue). See Supplementary Fig. 6A for bar graph representation of mean values across vigilance states. **C** Characteristics of sleep architecture in WT and KO^NAC^ mice baseline sleep (BL) and during SR. (Left): Average number of episodes per h for each vigilance state. Wake: [WT BL] - [WT SR] *P = 0.018; [WT BL] - [KO^NAC^SR] **P = 0.007; [KO^NAC^] - [WT SR] *P = 0.011; [KO^NAC^] - [KO^NAC^ SR] **P = 0.003; NREM: [WT BL] - [KO^NAC^ SR] *P = 0.037; [WT BL] - [KO^NAC^ SR] **P = 0.038; [KO^NAC^] - [KO^NAC^ SR] **P = 0.022. (Middle): Average episode duration for each vigilance state. Wake: [WT BL] - [WT SR] *P = 0.018; [WT BL] - [KO^NAC^ SR] **P = 0.007; [KO^NAC^] - [WT SR] *P = 0.011; [KO^NAC^] - [KO^NAC^ SR] **P = 0.003; NREM: [WT BL] - [KO^NAC^ SR] *P = 0.037; [WT BL] - [KO^NAC^ SR] **P = 0.038; [KO^NAC^] - [KO^NAC^ SR] **P = 0.022. (Right): Percentage of total time spent on each vigilance state: Wake: [KO^NAC^] - [WT SR] **P < 0.001; [KO^NAC^] - [KO^NAC^ SR] *P = 0.020; NREM: [WT BL] - [WT SR] *P = 0.017; [WT BL] - [KO^NAC^ SR] *P = 0.041; [KO^NAC^] - [WT SR] ^*^P = 0.011; [KO^NAM^] - [KO^NAM^ SR] *P = 0.048.; [KO^NAC^] - [KO^NAC^ SR] **P = 0.022, (WT *n* = 5 and KO *n* = 5). **D** Delta 2 (δ2) power (2.5–3.5 Hz) during NREM sleep at BL and during the first h of SR (F = 57.76 DFn = 3, DFd = 16) taken from the EEG and tetrode electrodes within: EEG^front^ [WT BL] - [WT SR] *P = 0.016, [WT BL] - [KO BL] *P = 0.04, [WT SR] - [KO BL] **P = 0.001, [KO BL] - [KO SR] *P = 0.016; ACC [WT BL] - [WT SR] *P = 0.04 *P = 0.03, [WT SR] - [KO BL]*P = 0.02, [KO BL] - [KO SR] *P = 0.04; AD [WT BL] - [WT SR] *P = 0.04, [WT SR] - [KO BL] *P = 0.02, [KO BL] - [KO SR] *P = 0.04; TRN [WT BL] - [WT SR] *P = 0.04, [WT SR] - [KO BL] **P = 0.008; *n* = 5 WT, 5 KO^NAC^. **E** Spindle amplitude during BL and SR NREM sleep. **F** Spindle rate during BL and SR NREM sleep (F = 76.15 DFn = 3, DFd = 16) in: EEG^front^: [WT BL] - [WT SR] **P = 0.002, [WT SR] - [KO BL] ***P < 0.001, [WT SR] - [KO BL] *P = 0.05, [KO BL] - [KO SR] *P = 0.02; ACC [WT BL] - [WT SR] *P = 0.04; AD [WT BL] - [WT SR] ***P < 0.001, [KO BL] - [KO SR] **P = 0.005; TRN [WT BL] - [WT SR] ***P = 0.004, [KO BL] - [KO SR] **P = 0.008. **G** Spindle length during BL and SR NREM sleep. **H** Comodulograms of phase amplitude coupling between ACC and AD in WT and KO^NAC^ during BL and SR. **I** Circular plots of averaged phase coupling between SW phase of one cortical of thalamic nuclei (first pair) and spindle amplitude of another cortical of thalamic nuclei (second pair) during the baseline (black line) and SR (blue line) of KO in EEG front (^front^) and parietal (^par^) and the ACC - AD. See Supplementary Fig. 6G for phase distribution in all locations. **J** Modulation index during BL NREM sleep and SR. No significant differences between phenotypes and conditions except for interactions between EEG^front^ - EEG^par^ for [WTBL] - [KO SR] *P = 0.45, t = 4.075 DF = 5.62 and ACC-AD [KO BL] - [KO SR] ***P < 0.001, t = 6.502, DF = 3.65, WT *n* = 5 and KO *n* = 4 for both BL and SR. **K** Spike rate of isolated cells during BL NREM sleep in ACC (*n* = 4, 3); AD (*n* = 3, 3) TRN (*n* = 3, 3); Brr (*n* = 2, 5) and VPL (*n* = 3, 5) of WT and KO, respectively. The insert shows the spike rate of cells isolated from WT and *Gclm* KO without NAC treatment (extracted from Fig. 4C). Significance levels were calculated using two-way ANOVA with Bonferroni’s multiple comparisons test. All results are represented by the mean + /−s.e.m.

Finally, we tested whether the administration of N-acetylcysteine (NAC), an antioxidant reported to mitigate symptoms and cognitive deficits in schizophrenia patients [75, 88], can rescue the fragmented sleep and the lack of homeostatic response in *Gclm* KO mice (Fig. 5). In these mice, *per oros* NAC administration has been shown to abolish OxS, normalize PV + immunoreactive neurons and the associated perineuronal net in both ACC and TRN [77, 80] and prevent hypofunction of T-type calcium currents in TRN neurons [77]. Importantly, we found that NAC-treated *Gclm* KO mice exhibited a sleep-wake cycle architecture in both BL and SR conditions indistinguishable from control mice (Fig. 5A-C and Supplementary Fig. 6A), indicating that NAC rescued the sleep fragmentation observed in non-treated *Gclm* KO mice (Fig. 1F–I). Furthermore, and contrary to non-treated KO mice (Fig. 2E), NAC-treated KO mice showed a significant upregulation of δ2 power during SR (compared BL) in EEG^front^, ACC, AD and VPL (Fig. 5D and Supplementary Fig. 6B). Moreover, NAC fully normalized the amplitude and duration of spindles, and reinstated a typical homeostatic upregulation of the spindle rate during SR (Fig. 2F-H versus Fig. 5E-G and Supplementary Fig. 6C-E). The coupling between SWs and spindles in all recorded regions were alleviated except for frontal cortex and AD during the SR (comparison with the non-treated WT animals; Supplementary Fig. 6F). Similarly, the abnormal high level of SW-spindle PAC between cortical areas and within non-sensory TC circuits in *Gclm* KO during SR (Fig. 3D) was partially abolished by NAC. This modulation was normalized between ACC and Brr and to a lesser extent between TRN and AD, but not between ACC and AD (Fig. 5H-I and Supplementary Fig. 6G). Finally, supporting these results, neuronal spike activity was rescued by NAC treatment in ACC, AD and TRN *Gclm* KO mice (Fig. 5K and Supplementary Fig. 6H-I). These results showed that NAC treatment in adulthood ameliorated sleep architecture and homeostasis, and normalized neuronal activity and network dynamics within non-sensory TC networks.

## Discussion

In this study, we investigated for the first time the impact of a redox dysregulation / OxS on sleep and the dynamics of thalamocortical brain regions known to regulate sleep-wake state switching and sleep homeostasis [19–21, 29]. We addressed this using multisite in-vivo electrophysiological recordings in freely-behaving *Gclm* mice, a mouse model prone to OxS due to a reduced capacity of GSH synthesis. EEG analysis of *Gclm* KO mice revealed a significant fragmentation of the sleep-wake cycle evidenced by an increased number, and shorter duration, of NREM sleep and Wake episodes during both the inactive (light) and active (dark) periods. During the SR following SD, the latency to first NREM sleep episode, SWA and spindle rate were all impaired in *Gclm* KO animals during SR period, suggesting an impairment of sleep homeostasis. This was accompanied by a reduced local coupling between slow waves and spindles and an increase of phase-amplitude coupling between SW and spindles within non-sensory TC circuits. Importantly, NAC pharmacological treatment rescued previous sleep fragmentation and sleep spindle deficits observed during SR in *Gclm* KO mice suggestive of a greater susceptibility of frontal TC circuits to OxS. Altogether, these findings provide new evidence of OxS-dependent dysregulation of local cellular activity in high-order thalamic networks that may contribute to the disturbances of fronto-parietal oscillatory activity during sleep in SZ.

### Sleep-wake cycle regulation

Sleep fragmentation in *Gclm* KO mice was characterized by an increased number, and shorter episodes, of wakefulness and NREM sleep while the REM sleep episodes remained unchanged, suggesting a selective alteration of the neural mechanisms of NREM sleep. Beside the extra-thalamic circuits regulating NREM sleep [89], transitions between wakefulness and NREM sleep are regulated by the firing modes of neurons within frontal TC circuits (ACC, AD, and mediodorsal thalamus (MD)) [46, 90, 91] and local thalamic circuits implicating the TRN [25, 27, 29, 52]. On this regard, *Gclm* KO mice showed a reduced number of PV + immunoreactive cells and abnormal perineuronal net in TRN and ACC [92] which was further confirmed in the present study. Thus, abnormal function of inhibitory neurons in these two TC circuits is likely to contribute to the sleep fragmentation and altered NREM sleep oscillations observed in this study.

Interestingly, compared to WT mice, the spike rate and bursting activity were higher in ACC of *Gclm* KO mice during wakefulness and NREM sleep. Conversely, TRN neurons of *Gclm* KO were less active, with less bursting activity and shorter bursts. This was accompanied with a reduced spindle length [58] in the TRN and AD. These results are consistent with ex-vivo data showing low excitability and higher threshold for burst firing associated with reduced function of T-type calcium channels in TRN neurons from *Gclm* KO mice [76, 77]. Whether TRN cell response to subcortical inputs, and entrainment of spindles, are causally involved in these pathophysiological mechanisms await further investigation.

As the firing pattern of of TRN neurons modulate SWA, the expression of spindles and the synchronization between slow waves and spindles during NREM sleep [25, 29, 33, 93–95], we expected a possible alteration of delta and/or spindle rate. While δ2 power was affected – which depends on frontal TC circuits [36] - spindle rates were similar in *Gclm* KO and in control mice during BL NREM sleep. Nevertheless, the reduced duration of the TRN cell bursts in *Gclm* KO mice is consistent with the shorter spindle duration, as suggested by previous study [44]. Importantly, NAC antioxidative normalized the neuronal firing rate in ACC and TRN during baseline NREM sleep, spindle amplitude and length of spindles (Fig. 5E, G and Supplementary Fig. 6C,D, H,I), further supporting an OxS-mediated alteration of frontal TC networks that directly alter sleep regulation and sleep oscillations.

### Impaired sleep homeostasis and TC network dynamics

The latency to sleep during light phase was unaffected in *Gclm* KO mice, but reduced during the dark phase and extended after SD. This points towards an impairment of sleep homeostasis rather than alteration of circadian processes. In both mice [26] and humans [96], the generation and homeostatic modulation of SWA and the propagation of SWs from frontal to parietal regions require functional frontal TC networks, including the mediodorsal thalamus (CMT), the ACC and the anterior thalamus (e.g., AD, AM or AV). Here, we found that *Gcml* KO and WT mice did not exhibit the typical increases in δ2 and spindle rate during SR as observed in WT mice. This is consistent with the alteration of frontal TC network dynamics. Conversely, δ1 activity is relatively insensitive to sleep pressure [36] and was increased in the sensory-TC circuits (Brr and VPL, Supplementary Fig. 2B) of *Gclm* KO mice, further suggesting that δ1 and δ2 oscillations recruit different brain networks.

The aberrant SD-induced modulation of SWA and atypical spindle expression (i.e., lack of increase in amplitude and decreased in length) in the AD, Brr cortex and TRN in *Gclm* KO mice may thus be caused by abnormal inhibitory or excitatory balances within local circuits. Our findings suggested that changes in the neuronal firing may reflect the absence of tuning of the instant firing frequency of isolated neurons at low frequency oscillations, which may be accounting for the decrease in length and increase in amplitude of spindles. At the microcircuit level, co-occurrence between SW and spindles is associated with high concurrent activity of pyramidal neurons and PV interneurons in the cortex [97]. Despite the recovery of the spiking rate in NAC treated animals, ACC recorded neurons showed a downward trend relative to controls that together with the overreaching spindle amplitude in the AD may reflect the dysregulation of inhibitory/excitatory neuronal activity and explain the lack of full recovery in the CFC at frontal EEG and AD. Additionally, TC feedforward network circuits, cortico-thalamic feedback loops and other circuits (i.e., brainstem) may be implicated in modulating local circuit dynamics, as previously suggested [58, 98] (see Supplementary Fig. 7). Investigation of the activity levels of interneurons and pyramidal cells would be valuable to fully understand the underlying mechanisms.

Changes in the local temporal organization of these oscillations may also affect the overall synchronization of TC networks. To assess this, we estimated the long-range modulation of the amplitude of spindle oscillations in one region of the TC networks by the phase of slow waves in a distant but connected TC area. Such phase-amplitude coupling (PAC) has been associated with cognitive and sensory processing in humans [99, 100] and rodents [101–104] and with altered procedural and emotional processing in SZ patients [105–107]. PAC analysis revealed an enhanced SW-spindle oscillation in non-sensory TC networks (TRN - AD, ACC - AD) and within cortico-cortical networks (EEG^front^ - EEG^par^; ACC - Brr) of *Gclm* KO during SR. Given that slow waves dynamics across the brain occur from frontal to posteriorly across the cortex [96], and spindles organize from the centroparietal regions to frontal cortices [39, 96, 108, 109], changes in the modulation indexes in KO may mirror variations in the amplitude of spindles or the phase coupling between slow waves and spindles from different brain regions. Based on the present data, our results suggested that the amplitude of spindles may be affecting the dynamics between non-sensory TC networks. However, changes in the phase coupling could contribute to the increase in cortico-cortical networks activity. In this context, PV + interneurons receive a large proportion of thalamic inputs, preferential from high order thalamus and control feed-forward inhibition of pyramidal neurons that in turn exert top down modulation of thalamic nuclei [110–113] (Supplementary Fig. 7). During SR of *Gclm* KO mice, changes in the inhibitory tone of PV interneurons within the ACC and TRN but nor in the Brr [65] may provide a weak temporal constraint on long-range synchronization of SWs and spindles. Particularly in circuits vulnerable to OxS as previously reported [114–116]. Although NAC normalized the modeulation index between some of these long-range connections, it did not abolish the aberrant synchrony between EEG front-par and ACC-AD remained, possibly due only a partial re-normalization SW spindle phase co-occurrence (Fig. 5J) and the spindle amplitude detected in the AD and ACC (Fig. 5E, K). Further experiments addressing different time points of NAC administration may be helpful to determine the critical points for phenotype conversion. Overall, these results support the original hypothesis that *Gclm* KO mice are vulnerable to SD, as well as to oxidative challenges [80], yet further investigation of this phenomenon in conditional KO mice will be informative on the cellular mechanisms involved.

### Redox status and sleep

Redox status, oxidative stress and cytokines levels, which are typically modulated by sleep pressure, represent key signals of sleep homeostasis. In *Gclm* KO mice, dysregulation of systemic redox and oxidative stress, together with high levels of cytokines may thus affect sleep homeostasis. OxS builds up during wakefulness [70, 71] and dissipates during the subsequent sleep. Furthermore, extended periods of SD drastically increase OxS [70, 117] that contribute to the consequent memory impairment [118]. Due its impaired GSH system, *Gclm* KO mice cannot efficiently neutralize OxS induced by mild SD, thus further exacerbating the deleterious effects of OxS on normal functioning of TC network, possibly via further PV + neuronal impairments. The excess of SD-induced OxS coupled with a need for increased metabolic demand imposed on fast-spiking PV + interneurons in ACC but also in the TRN neurons could possibly explain the failure of *Gclm* KO mice to display a proper homeostatic response with increase in spindle rates and the nesting of spindles within SWs in ACC. Interestingly and contrary to the ACC, the somatosensory cortex did not show any increase in spindle nesting within SWs in WT mice during SR. Combined with the fact that PV + neurons in the somatosensory cortex of *Gclm* KO mice are less susceptible to oxidative challenge than those in the ACC [80], this may explain why *Glcm* KO mice displayed stronger functional abnormalities in non-sensory, rather than sensory, TC networks. Yet, the differences in thalamocortical and cortico-thalamic wiring within non-sensory (high order) and sensory TC circuits [119, 120] may also influence the respective vulnerability of these different TC networks to OxS. This circuit specific sensitivity to OxS may explain the partial pharmacological rescue of δ2 oscillations and further suggest possible developmental influences. Further investigation is warranted for dissecting developmental specific cell-type alterations responsible for the various dysregulations of TC network dynamics found in these mice.

### Contribution of OxS to sleep-related anomalies in SZ patients

Redox dysregulation and OxS are among the pathological processes linked to schizophrenia that arise independently from a variety of genetic and environmental factors affecting various biological systems, ranging from metabolism to neuroinflammation and glutamatergic neurotransmission [68]. Subsets of subjects with SZ displayed brain GSH deficit possibly resulting from either a compromised GSH system due to genetic or epigenetic origins, or a failure to maintain proper redox regulation in the brain [121]. In this regard, *Gclm* KO mice are a relevant model to investigate the OxS contribution to some of the pathological mechanisms of SZ. Results from the present study indicated that OxS alone cannot recapitulate every aspect of the sleep disturbances and sleep-related EEG anomalies reported in human subjects with SZ. Still, OxS may contribute to abnormal sleep architecture, particularly in relation to NREM sleep regulation [122, 123]. Likewise, the lack of proper sleep homeostasis that together with sleep disturbances have been found in subjects with SZ [2, 19, 124] are likely linked to impaired mechanisms of OxS neutralization. While in Gclm KO mice, OxS mimics the reduced duration of spindles as reported in SZ [125], it does not recapitulate other pathological phenotypes. This suggest that other compensatory processes and possibly additional environmental insults during sensitive developmental period may play a role to fully express the heterogeneity of SZ. This is indeed highlighted both in early psychosis [126] and animal models [80].

The understanding of the mechanism underlying sleep-related disturbances in SZ remains poorly investigated. Surprisingly, only a small number of studies have investigated the sleep architecture in animal models relevant to SZ [127–130]. The sleep-related anomalies described in these different SZ rodent models diverge to some extent, suggestive of the involvement of multiple pathological mechanisms and heterogeneity of the sleep disturbances among subjects with SZ. Thus, a rare de novo mutation of the gene encoding the t-type calcium channel, Cav3.3 that is associated with SZ, does not affect sleep architecture, but decreases spindle density and length and increases delta oscillations during sleep [131]. On the other hand, GluA1 KO mice display increased sleep latency, longer REM duration episodes, spindle deficits, but enhanced homeostatic regulation of delta oscillations [127]. Finally, a neurodevelopmental model pertinent to psychosis, the prenatal methylazoxymethanol acetate (MAM) rodent model, shows fragmented sleep characterized by reduced duration of NREM episodes, small decreased in spindle density, and disrupted coupling between delta and spindles in posterior cortical areas [132]. Interestingly, this later model has several features common to *Gclm* KO mice, including the presence of OxS, abnormal PV + immunoreactive cells and perineuronal net in ACC and TRN and reduced T-type calcium currents in TRN neurons [77].

## Conclusion

The present study, which is to our knowledge the first one investigating sleep-related TC network dynamics in a rodent model pertinent to SZ, describes circuit mechanisms responsible for alterations in sleep architecture, sleep oscillations and sleep homeostasis found in some subjects with SZ. Growing body of literature highlights the potential of sleep as a window for neuromodulation therapy in neurological and neuropsychiatry disorders. Identification of temporal and topographical alterations in neuronal activity are thus essential to select proper targets for neuromodulation. Recent investigations both in humans [60, 133] and animal models [134, 135] have suggested procedures to enable real-time tracking and manipulation of sleep EEG oscillatory activity using closed-loop transcranial stimulation or auditory stimulation to enhance or diminish SWs and spindles and to facilitate brain plasticity and repair [133, 136, 137]. Our work highlights the TRN - AD - ACC thalamocortical circuits as a potential targets for future neuromodulation approaches. Furthermore, the beneficial effect of NAC on most sleep-related abnormalities in *Gclm* KO mice points to the need for assessing sleep quantity (architecture of the sleep-wake cycle) and sleep quality (oscillations) during clinical trials using antioxidant compounds in subjects with schizophrenia. Ultimately, promoting the characterization of the neuronal network dynamics and sleep oscillations may provide new insights in the prediction, diagnostic, or prognostics as biomarkers for different types of SZ and other psychiatric disorders.

## Supplementary information

Supplementary material and methods

supplementary materials and methods

Supplementary figures and figure legends

Supplementary figures

Supplementary Figure 1

Supplementary Figure 2

Supplementary Figure 3

Supplementary Figure 4

Supplementary Figure 5

Supplementary Figure 6

Supplementary Figure 7

Supplementary Table 1

## Data Availability

All data and scripts are available upon request.
