# Supplementary material for: Alterations in TRN-anterodorsal thalamocortical circuits affect sleep architecture and homeostatic processes in oxidative stress vulnerable Gclm−/− mice
Source: Mol Psychiatry. 2022 Jul 28;27(11):4394–406. doi: 10.1038/s41380-022-01700-w (PMC9734061; doi:10.1038/s41380-022-01700-w)
Supplement: Supplementary file 2 — supplementary materials and methods [file 41380_2022_1700_MOESM2_ESM.pdf]

Animals

Only adult male mice were used in the experiments. Animals were housed in IVC cages in groups of 2–5 before instrumentation. After implantation, all mice were housed individually. Animals were habituated to the recording cables in their open-top home cages (300 × 170 mm) and kept tethered for the duration of the experiments. Animals were allowed to move freely in the cages during *in-vivo* electrophysiology experiments. Before commencing experimental recordings, baseline sleep was recorded and compared to previously published results [1–3]. Experiments were performed during the "light phase", the "sleep recovery period" (12:00 (ZGT4)–16:00 (ZGT9)) and the" dark phase" (24:00 (ZGT16)-04:00 (ZGT20)). All recordings were performed from 16-20 weeks of age. Animals with heterozygote genetic background were excluded. We included 3 different cohorts of animals for the initial study on *Gclm* WT and *Gclm* KO, and 2 cohorts of *Gclm* WT and *Gclm* -/- treated with NAC for the rescue experiment. *Gclm* KO mice for all experiments were selected randomly among their litter mates. Experimenters were blinded to the genotype and to the treatment with NAC or water.

Instrumentation

Animals were anaesthetized with a multimodal balanced protocol consisting in an injection of fentanyl (0.05 mg/kg; Fentanyl, Kantonsapotheke Zurich, CH), midazolam (5 mg/kg; Dormicum®, Roche Pharma AG, Reinach, CH) and medetomidine (0.5 mg/kg; Dorbene®, Graeub AG, Bern, CH) mixed in NaCl (0.9%) to a volume of 3 µl/g body weight. Intraoperative analgesia was provided by a local anaesthesia of lidocaine (Xylocaine 2%, diluted 1:1 in sterile NaCl 0.2 mL/site) on the incision site. Saline 10 ml/kg and meloxicam 5 mg/kg were given subcutaneously. The skin on the head was shaved and aseptically prepared. A single longitudinal midline incision was made from the level of the lateral canthus of the eyes to the lambda skull suture. Two stainless steel screws were placed in the skull to measure EEG (frontal EEG: AP −2.0 mm, ML +2.0 mm, parietal EEG: AP −3.0 mm, ML +2.7 mm) and a third stainless steel screw was place in the cerebellum as a reference. Two bare-ended wires were sutured to the trapezius muscle on each side of the neck to record EMG. Tetrodes were made from four strands of 10 µm twisted tungsten wires with 100-200KΩ, connected to an electrode interface board by gold pins. Individual tetrodes were secure to a tetrode holder (in house design, 3D printed) fixed into the stereotaxic frame. Tetrodes were stereotaxically placed (30µm/ s) ipsilaterally and individually in the following order: 1) anterior cingulate cortex (ACC: AP +1.2 mm, ML +0.2 mm, DV −1.5 mm); 2) anterior dorsal thalamus (AD: AP −0.86 mm, ML +0.75 mm, DV −2.75 mm), 3) the reticular thalamic nucleus (TRN: AP −0.8 mm, ML +1.7 mm, DV −3.5 mm), 4) ventral posterolateral nucleus (VPL: AP −1.6 mm, ML −1.82 mm, DV −3.6 mm) and 5) the sensory cortex (Brr: AP −1.7 mm, ML +2.8 mm, DV −1.0 mm). Dental acrylic (C&B Meta–bond) was applied around each tetrode after implantation with a waiting period of 15 min between tetrodes. Finally, once all tetrodes were implanted, the tetrode bundle and the electrode interface board were placed with the long edge of the board perpendicular to the middle line and on the posterior part of the mouse head. The implant was stabilized using a methyl methacrylate cement. Animals were allowed to recover in

their home cage over a heating mat for 48 hours. A total recovery period of a minimum of 5 days was allowed for recovery before tethering. Animals were tethered using a 32 channel RHD2132 headstage (intan technologies, part #C3314) connected to an SPI interface cable (intan technologies, part #C3206) 7 days before starting recordings. Habituation to the cables was performed progressively from 1 hour the first day, 4 hours the second day, 8 hours the third day and kept plugged for the rest of the recording period. Animals were handled 10 min per 3 consecutive days prior tethering to the cables.

In-vivo electroencephalographic recordings

For all electroencephalogram (EEG), electromyograms (EMG) and local field potentials (LFP)- tetrode recordings, mice were connected to an RHD USB interface board (Intan Technologies, part #C3100) and an open source software (RHD2000 evaluation software, Intan Technologies). Data was sampled at 20 kHz. Baseline recordings started 3 days after animals had nested and resumed a normal sleep–wake cycle. All baseline and recovery sleep recordings were performed between Zeitgeber time 12:00 (ZT4) to 16:00 (ZT8). Sleep deprivation was performed using the gentle handling (GH) procedure where the experimenter introduced objects to the home cage of the animal to keep the mouse awake for an extended period 4 hours (from the time of lights on, when animals start their sleep period), corresponding to the ZT0 to ZT4. The procedure was carried out with minimal interaction with the aim to reduce the spurious effects of stress and of forced locomotor activity. GH was performed under the constant physical presence of a fully trained experimenters with whom animals were familiarized (see above section) and were actively monitored by the experimenter with the support of online EEG and electromyographic recordings.

Histological characterization and immunohistochemistry

For confirmation of electrode placement, animals were deeply anaesthetized with isoflurane 5% for induction and 1.5% for sustenance. Electrolytic lesions were made by passing an anodal current (30 μA for 10 s) through each tetrode, followed by a recovery period of 2 hours. Then animals were injected with 15 mg pentobarbital (i.p.) and transfused over the heart with 20 ml ice cold heparinized PBS followed by 30 ml 4% formalin. Brains were removed and post-fixed overnight in 4% formalin. They were then cryoprotected in 40% sucrose for 24–48 hours. Brain sections (30-μm thick) were prepared with a cryostat. The exact position of each tetrode was determined using Nissl-staining with Cresyl violet and mouse atlas Paxinos and Frankling 2004 [4]. 1:3 series of brain slices were processed for the detection of fluorescence and quantification of immunopositive PV+ cells. Free-floating sections were washed in PBS plus 0.1% Triton X-100 (PBS-T) three times for 10 minutes each, and then blocked by incubation with 10% normal donkey serum (NDS) in PBS-T for 1 hour. Free floating sections were incubated with primary antibodies for PV (Abcam, Cat. No. 11 427; 1:500) for 24–48 hours at 4 °C in blocking solution containing 2% NDS. Sections were then washed in PBS-T, three times for 10 min each, and then incubated with secondary antibody (Abcam: AB96947, 1:500) for 1 hour at room temperature. Slices were then mounted on glass slides and coverslipped with

Fluoromount-G (0100-001, Southern Biotech) and allowed to dry. Slides containing labelled brain sections were imaged using a Nikon-PLAN Fluor 20x/0.3NA objective on a Nikon Eclipse Ti-E Fluorescence microscope controlled by Nikon NIS software. Fluorescent signals were normalized by the background fluorescence levels per individual slide to ensure proper comparison across sections and animals. 8 bit images were automatically thresholded and a same size region of interest (ROI) used to delineate PV^+ immunopositive individual neurons in a squared area of 250 μm^2 using ImageJ software. The total number of manually ROIs delineated PV cells was analysed using ImageJ software and the particle analysis toolbox. Sections (anterior-posterior) containing either the ACC (from bregma +1.5 - 0.8) or the TRN (from bregma -0.5 to 1.3) were selected for the analysis. Three sections per brain region and animal (3 mice per genetic background) were included for the analysis (Figure 1A).

Determination of vigilance state

We define wake episodes as periods of low amplitude EEG. Prominent theta band EEG activity and concurrent high amplitude EMG activity, corresponding to bursts of movement-related activity, and arousals shorter than 1 second were disregarded. Periods of low EMG tone with characteristic EEG and theta activity were scored as wakefulness including feeding and grooming behaviours. We defined NREM sleep as periods with a relatively high amplitude rich in low-frequency EEG and reduced muscle tone relative to wakefulness associated with behavioural quiescence. We scored REM sleep as sustained periods of theta band EEG activity and behavioural immobility associated with muscle atonia with brief phasic muscle twitches. Data were scored independently by two experimenters.

Analyses

Data analyses were carried out using custom scripts written in MATLAB® (R2018b, MathWorks, Natick, MA, USA). Furthermore, built-in functions from Wavelet and Signal Processing toolboxes of MATLAB were used as described below.

Detection of spindles using an automated algorithm

Spindles were detected during NREM sleep using an optimized wavelet-based method, as previously published[31]. In brief, we estimated the wavelet energy of LFP/EEG signals using the complex B-spline mother wavelet within 9-16Hz, smoothed it using a 200 ms Hanning window, and then applied a threshold equal to 3 SD (SD: standard deviation) above the mean to detect the potential spindle events. A lower threshold of 1 SD above the mean was set to find the start and the end of detected events. Events shorter than 400 ms or longer than 2 s were discarded. Using band pass-filtered LFP signals in the spindle range (10–16 Hz), we automatically counted the number of cycles of each detected event and excluded those with <5 cycles or more than 30 cycles. To discard artefacts,

events with a power in the spindle band lower than 6–8.5 Hz or higher than 16.5–20 Hz power bands were not included.

Quantification of spindle rates during vigilance state transitions

We estimated spindle rate before state switching from NREM to REM and wake, separately. We first marked all NREM–REM and NREM–Wake transition points, which were scored with 1 s resolution. Spindle rate was calculated by the average of all spindles occurring in all NREM that were at least 25 s or longer. Then, the total number of detected spindles were divided by the time per episode. Graphs report the average per animal. Spindle length was calculated taken from the beginning of the detected Spidle where the filtered signal crosses the lower limit threshold till the end determined by the time where the LFP signal crosses at the down facing crossing of the limit threshold (see figure 2). We averaged over all transitions for each animal to obtain the spindle rate per animal, then we averaged the values obtained from all animals and compared per condition (Figure 3 and Suppl. Figure 3).

Cross-correlation between slow waves and spindles. First, LFP/EEG signals were bandpass filtered in 0.5–4 Hz for SWs and 10-16 Hz for spindles, using an order equal to three cycles of the low cutoff frequency (6000th and 333rd order respectively) using window-based finite impulse response (FIR) filters in both the forward and reverse directions to eliminate phase distortion (“filtfilt” function, MATLAB). Then, envelopes of spindles were extracted using the Hilbert transform. Both SWs and spindle envelopes were then aligned to the start of the detected spindles (see above for description), and averaged across entire NREM sleep episodes. To quantify the relation between the timing of occurrence between SWs and spindles locally in different thalamocortical regions, cross-correlation values were estimated by calculating the normalized power between averaged signals of SWs and spindle envelopes detected during NREM sleep episodes in a 4 h period.

Phase locking slow waves (SW) and spindles

SW and spindles were detected as described above and by [1]. Start and end of each individual event were computed. We correlated the time on which SW (first selected pair) from one brain area will coincided with spindle event in another brain area (second pair). Event with inter-event intervals < 0.2 s were not considered. LFP signals were filtered by applying the Hilbert transform. Filtered LFP signals were used to extract the angle at each sample (time) to get a continuous representation of the relative SW/spindle phase. To assess the absolute phase across a SW the relative phase was unwrapped and centered such that the phase was at the peak of the spindle and 50 ms before and after the peak. In the case of the spindles we included spikes occurring at each of the spindle cycles between the start and end times of the spindle. For each spindle epoch and each unit, the nearest phase was collected at each spike event. A polarized histogram was generated with the *18* number of bins represented in degrees. Each phase value in this distribution was treated as a vector of magnitude 1 and angle equal to vector generated by the mean Cosine and Sine. From this vector, we attained the phase-locking value (vector magnitude) and the preferred SW or spindle phase (vector

angle). We calculated these measures for each SW and spindle cycle per recording sessions per animal and averaged the values across all animals.

Modulation Index and Comodulogram analysis

We used the Modulation Index (MI) to measure phase–amplitude coupling (PAC) [39]. First, signals from four-hour recordings were filtered for SWs (0.5-4 Hz) and spindles (10-16 Hz) using FIR filters as described above. Then, we estimated the instantaneous phase of SWs and the envelope of spindle oscillations using the Hilbert transform. Then, we concatenated episodes of NREM sleep episodes to derive the vigilant stage-specific comodulogram graph. To avoid power line interferences, frequency bands in the vicinity of 60 Hz and its harmonics were reassigned with a 2-Hz safe margin from these interfering frequencies. We considered 18 frequency bands (20 degrees per bin) for phase (0.1 – 5 Hz, 1-Hz increments, 2-Hz bandwidth), and 28 frequency bands for amplitude (0.5–50 Hz, 1 Hz increments, 10 Hz bandwidth). Then, the phase of the SWs low was discretized into 18 equal bins (N = 18, each 20°) and the average value of detected spindle's envelope inside each bin was calculated. The resulting phase-amplitude histogram (P) was compared with a uniform distribution (U) using the Kullback–Leibler distance, $DKL(P, U) = \sum_{j=1}^{N} P(j) * \log[P(j)/U(j)]$, which was normalized by log(N) to obtain MI, $MI = DKL/\log(N)$. MI values were then calculated for all the pairs to obtain the comodulogram graphs.

Single unit analysis of spiking rate in vigilant state and state transitions.

Single units were considered for the analysis when their amplitude exceeded by 5 SD the noise. Smaller detected units were discarded. Then, we visually inspected sorted spikes and excluded from further analysis clusters of spikes with a completely symmetric shape (noise clusters), or with a mean firing rate < 0.2 Hz (see suppl. Figure 4A for an example of the detection and isolation of units). Averaged firing rate of each individual unit was calculated in each vigilant state as follows: the total number of spikes (action potentials) during a state divided by the total time spent in that specific state. Spiking rate is reported in (Hz). Only isolated spiking units that show modulation across vigilance states were included in the analysis. Burst firing of single units was determined as follows: minimum of three consecutive action potentials with inter-spike intervals (ISIs) < 6 ms, and preceded by a quiescent hyperpolarized state of at least 50 ms [1] . Spike transitions were computed by averaging the spiking rate activity during the last 5 seconds before transition to another vigilance state and the first 5 seconds of the subsequent vigilance state per recorded site using a 5 ms bin width. Neurons that showed modulation of spiking rate across vigilant state transitions (e.g. NREM to wakefulness) were excluded in the analysis. Histogram distribution of the transient spiking rates was generated using 0.5Hz bin size representing the probability of firing in each specific bin during NREM sleep. LFP-spiking rate relationships were obtained by calculating the LFP power magnitude every 0.5 Hz and plotted against averaged spiking of all detected units per location at that specific frequency.

Statistical methods

MATLAB® (R2018b, MathWorks, Natick, MA, USA) and Prism 8 (GraphPad) were used for statistical analysis. No power calculations were performed to determine sample sizes, but similarly sized cohorts were used as in other relevant investigations [1–3]. All data represents the average of individual averages per animal. Data were compared via two-way ANOVA mixed effect model for non-matched values followed by Bonferroni's multiple comparison test. All data was tested for normal distribution. Data comparisons test for parametric data using comparisons between phenotypes (WT vs KO) and the different experimental conditions (baseline sleep vs recovery sleep), as indicated in the text. Values in the text are reported as mean ± standard error mean (SEM). Significance levels represent *P< 0.033, **P< 0.002 and ***P< 0.001.

Figures were prepared in Adobe Illustrator CC (Adobe).

Bibliography

1. Bandarabadi M, Herrera CG, Gent TC, Bassetti C, Schindler K, Adamantidis AR. A role for spindles in the onset of rapid eye movement sleep. Nat Commun. 2020;11:5247.

2. Herrera CG, Cadavieco MC, Jego S, Ponomarenko A, Korotkova T, Adamantidis A. Hypothalamic feedforward inhibition of thalamocortical network controls arousal and consciousness. Nat Neurosci. 2016;19:290–298.

3. Gent TC, Bandarabadi M, Herrera CG, Adamantidis AR. Thalamic dual control of sleep and wakefulness. Nat Neurosci. 2018;21:974–984.

4. Paxinos G, Franklin K. The mouse brain in stereotaxic coordinates. 2004. 2004.
