# Supplementary material for: Alterations in TRN-anterodorsal thalamocortical circuits affect sleep architecture and homeostatic processes in oxidative stress vulnerable Gclm−/− mice
Source: Mol Psychiatry. 2022 Jul 28;27(11):4394–406. doi: 10.1038/s41380-022-01700-w (PMC9734061; doi:10.1038/s41380-022-01700-w)
Supplement: Supplementary file 3 — Supplementary figures and figure legends [file 41380_2022_1700_MOESM3_ESM.docx]

| **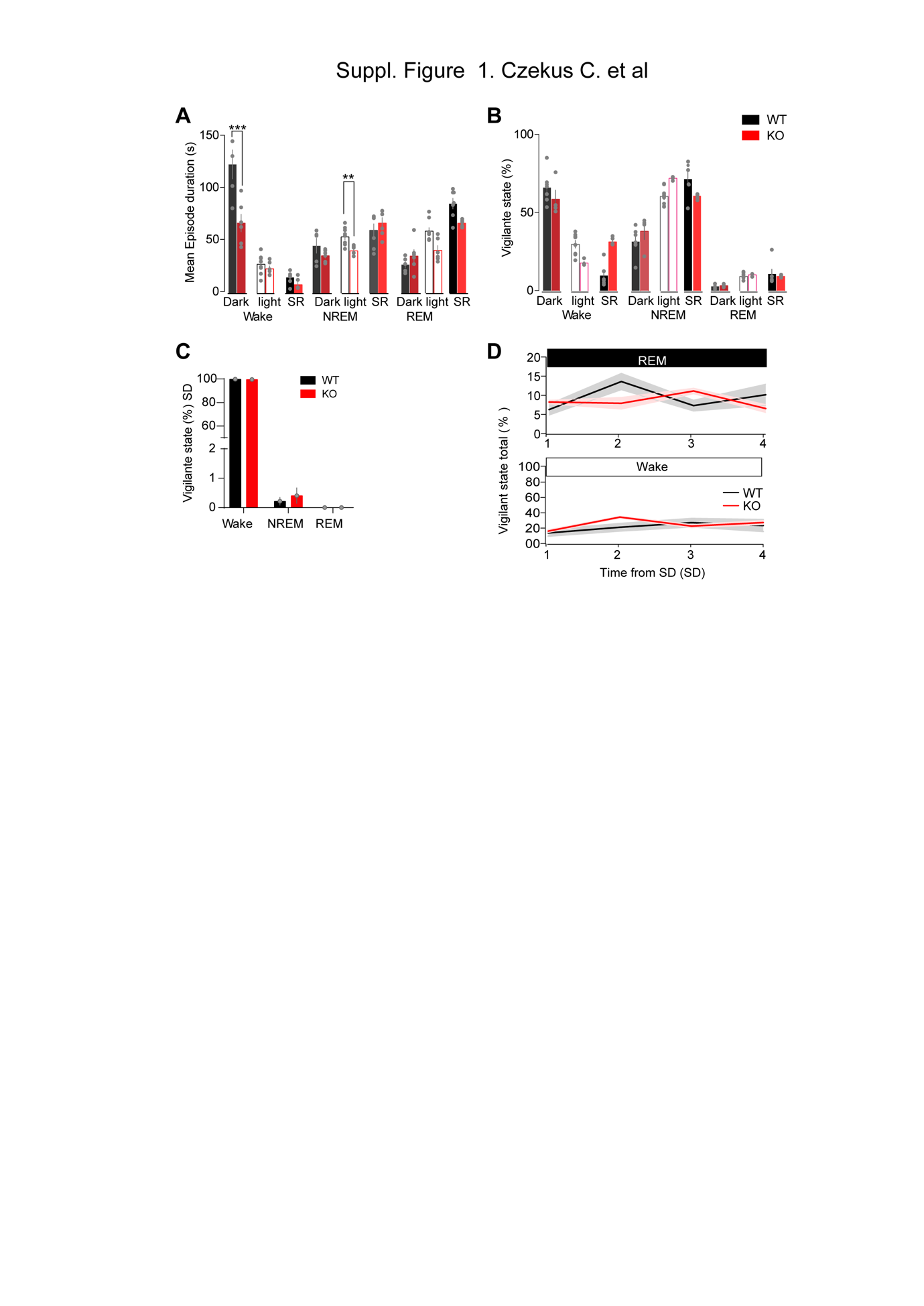** |
| --- |

**Suppl. Figure 1. Sleep characterization of *Gclm* WT and KO mice**. **A.** Episode duration of each vigilance state during respectively the dark and light (BL) phases, and the sleep recovery period (SR). Significance was found between genotype for Wake ([WT Dark - KO Dark] ***P< 0.001, t= 7.48) and NREM ([WT BL - KO BL] **P= 0.005, t= 3.074, DF= 98) Dark and light periods: n= 9, 6 for WT and KO respectively; n= 7 WT and n= 6 KO mice for SR period. **B.** Percentage of total of vigilance state during respectively the dark, light phase baseline (BL) and (SR) recorded from WT and KO mice. **C.** Quantification summary of the total amounts of wake, NREM and REM sleep spend during the SD protocol. Note that there was no REM expression (n= 7 WT and n= 6 KO mice). **D**. Progression of the percentage of wake and REM sleep during the first 4 hours of SR period after sleep deprivation (n= 4 and 5 WT and KO respectively). Significant levels were calculated using 2-way ANOVA and Bonferroni's multiple comparisons test. All data are represented by the mean +/- s.e.m values.

|  |
| --- |
| \| 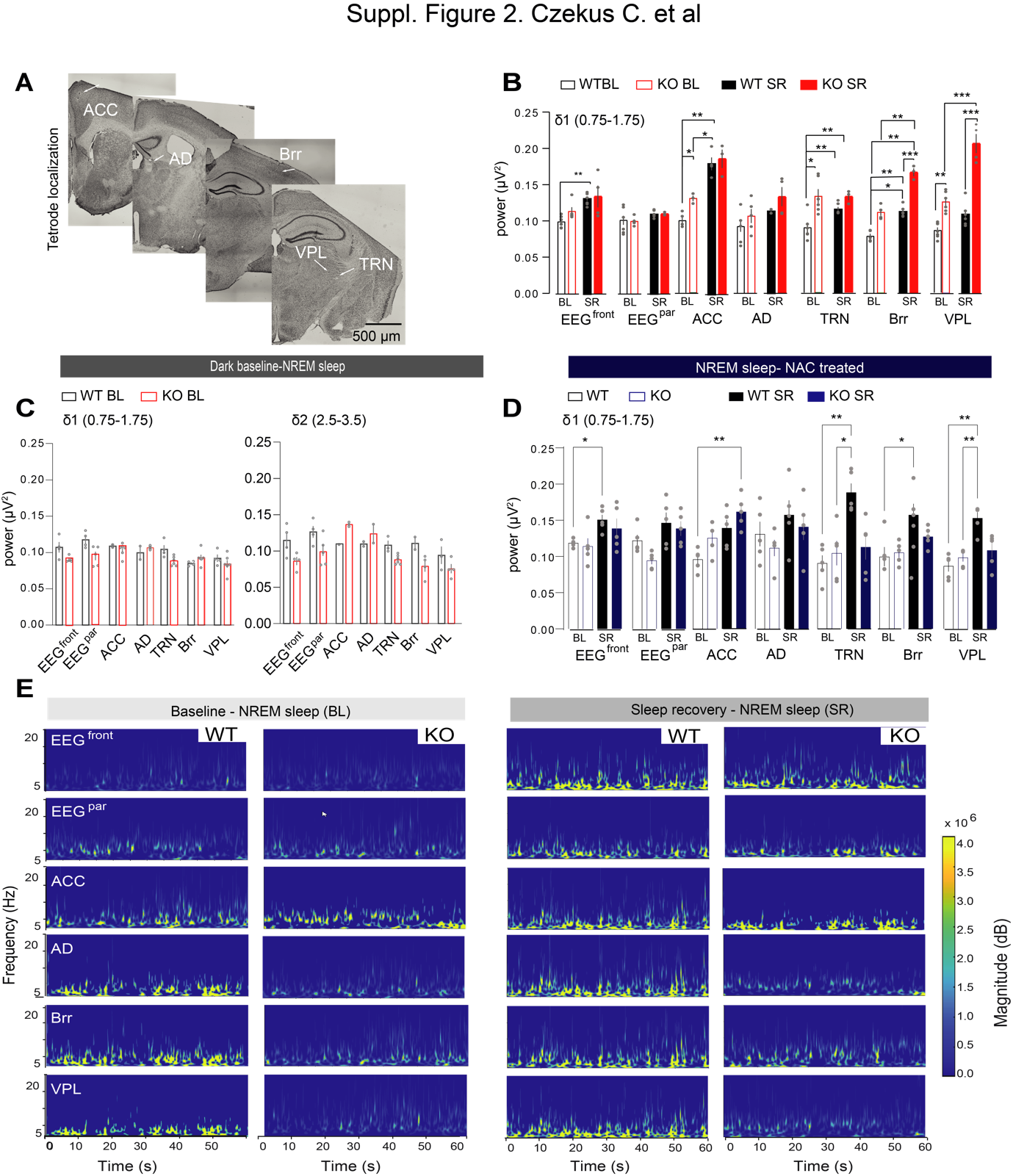 \| \| --- \| \| **Suppl. Figure 2. Changes in slow wave activity and delta oscillations in *Gclm* WT and KO mice**. **A.** Representative micrographs of cresyl violet staining showing the tetrode locations in the anterior cingulate (ACC), the anterior dorsal thalamus (AD), the reticular thalamic nucleus (TRN), the sensory cortex (Brr) and the ventral posterolateral thalamic nucleus (VPL). **B.** Normalized power of delta 1 (δ1, 0.75-1.75 Hz) during baseline (BL) and first h of sleep recovery (SR) NREM sleep filtered from the EEG and local thalamo-cortical LFP signals. Significance between groups F = 61.19 DFn= 3, DFd= 20; P***<0.001), significance per recording site: EEG^front^: [WT BL] - [WT SR] *** P< 0.001; ACC: [WT BL] - [KO BL] *P= 0.014, [WT BL] - [WT SR] **P= 0.005, [KO BL] - [WT SR] *P= 0.038; TRN: [WT BL] - [KO BL] *P= 0.020, [WT BL] - [WT SR] **P= 0.001, [WT BL] - [KO SR] **P= 0.008, Brr: [WT BL] - [KO BL] *P= 0.012, [KO BL] - [KO SR] **P<0.001, [WT BL] - [KO SR] **P= 0.002, [KO BL] - [KO SR] **P= 0.006, [WT SR] - [KO SR] *P= 0.013; VPL: [WT BL] - [BL KO] **P= 0.002, [WT BL] - [KO SR] **P= 0.010, [KO BL] - [KO SR] *P= 0.027, [WT SR] - [KO SR] *P= 0.010; EEG^front:^  BL n= 7, 5 and SR n=7, 4; EEG^par^ BL n= 7, 5 and SR n= 7, 3; ACC BL n= 7, 5 and SR n= 5, 4; AD BL n= 5, 1 and SR n= 2, 4; TRN BL n= 6, 6 and SR n= 6, 3; Brr BL n= 5, 4 and SR n= 6, 3; VPL BL n= 7, 6 and SR n= 6, 4 for WT and KO respectively. **C**. Power of delta 1 δ1, 0.75-1.75 Hz) and 2 (δ2, 2.5 - 3.5 Hz) during the dark cycle. EEG^front^ (EEG^front and par^: n= 4, 5; ACC: n= 2, 3; AD: n= 2, 2; TRN: n= 4, 3; Brr: n= 4, 3; VPL: n= 4, 3; for WT and KO respectively (F= 3.52 DFn= 1, DFd= 8, P=0097). **D.** δ1 values comparing WT and *Gclm* KO treated with NAC (*Gcml* KO^NAC^) showing significant between WT and KO (F= 20.45 DFn= 3, DFd= 16); significant increases in the BL and SR per recording site : EEG^front^: [WT BL] – [WT SR] *P= 0.047; TRN: [WT BL] - [WT SR] **P= 0.002, [WT SR] – [KO WT] *P= 0.028; VPL [WT BL] – [WT SR] **P= 0.002, [WT SR] - [KO BL] **P= 0.005, n= 5 WTs and n= 5 for KO both in BL and SR. **E.** Representative time-frequency of local power spectra recorded from EEG electrodes and tetrode electrodes within the TC networks in *WT* and *KO* animals during baseline (BL) (left) and sleep recovery (SR) (right). Data are represented by Statistical analysis was carried out using 2-way ANOVA and Bonferroni’s test for multiple comparisons.   \| 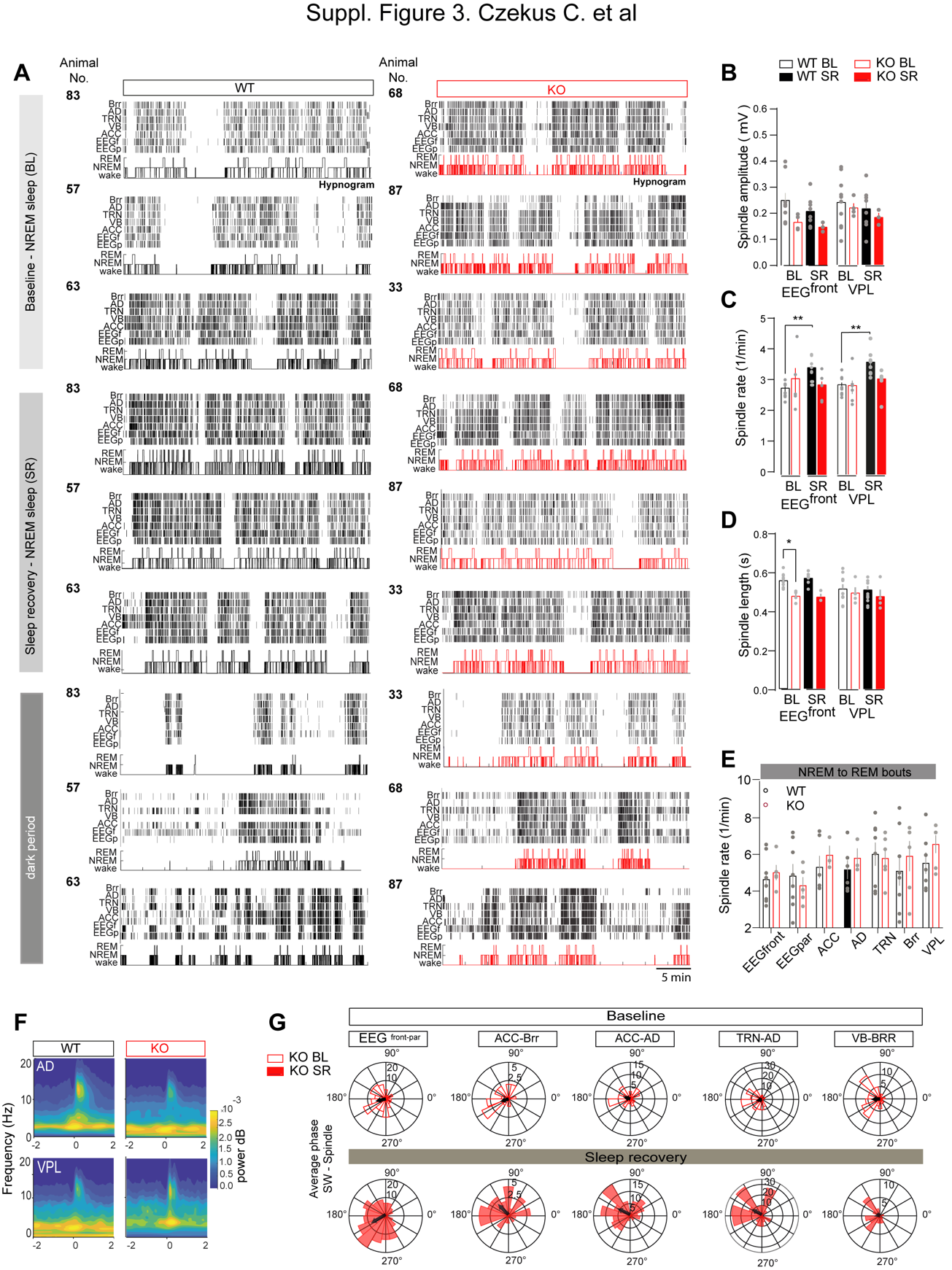 \| \| --- \| \| |

**Suppl. Figure 3. Spindle activity in *Gclm* WT and KO mice.** **A.** Representative detected (lines) spindle activity through the sleep wake cycle (denoted by the hypnogram on the bottom) during spontaneous occurring NREM sleep (light period), during the first hour of the sleep recovery (SR) and during the dark period. **B.** Spindle amplitude showing the individual values before (open bars) and after sleep deprivation during the first hour of sleep recovery (filled bars) in WT (black) and KO (red) mice BL n= 9, 4 and SR n= 8, 3 for WT and KO mice. **C.** Spindle rate calculated from EEG front [WT BL] - [WT SR]: **P< 0.007; EEG^par^: **P =0.007; VPL: [WT BL] - [WT SR]: **P= 0.008. Effect between BL and SR ***P< 0.001, F= 18.01 DFn= 3 DFd= 24.**D.** Spindle length. **H.** Spindle length during BL and SR NREM sleep: (effect ***P= <0.001, F= 18.22 DFn= 3, DFd= 24), EEG^front^: [WT BL] - [KO BL] **P= 0.003. Numbers for *B-D*, EEGs: BL n= 8, 6 and SR n= 8, 5 and for VPL: BL n= 8, 6 and SR n= 8, 6 for WT and KO mice respectively. **E.** Spindle rate calculated from the last 25 s of the NREM sleep till the transitions to REM sleep (EEG^front^: n= 8, 5; EEG^par^: n= 8, 5; ACC: n= 6, 3; AD: n= 6, 3; TRN: n= 8, 6; Brr: n= 8, 6; VPL: n= 7, 6 for WT and KO respectively). **F.** Representative time-frequency analysis of delta (0.5-4 Hz) and spindle envelop at the different recorded in AD (top) and VPL (bottom) for both WT and KO during NREM sleep BL. **G**. Circular representation the phase distribution of SW (first pair)-spindle (second pair) peak coupling between two brain areas (pairs) in WT (black line) and KO (red line) during baseline (top panel) and detected event during the NREM of the SR (bottom panel). Statistical differences were calculated using 2-way ANOVA and Bonferroni’s post hoc test to correct for multiple comparisons. Data are represented as the mean of the individual values per animal +/- s.e.m.


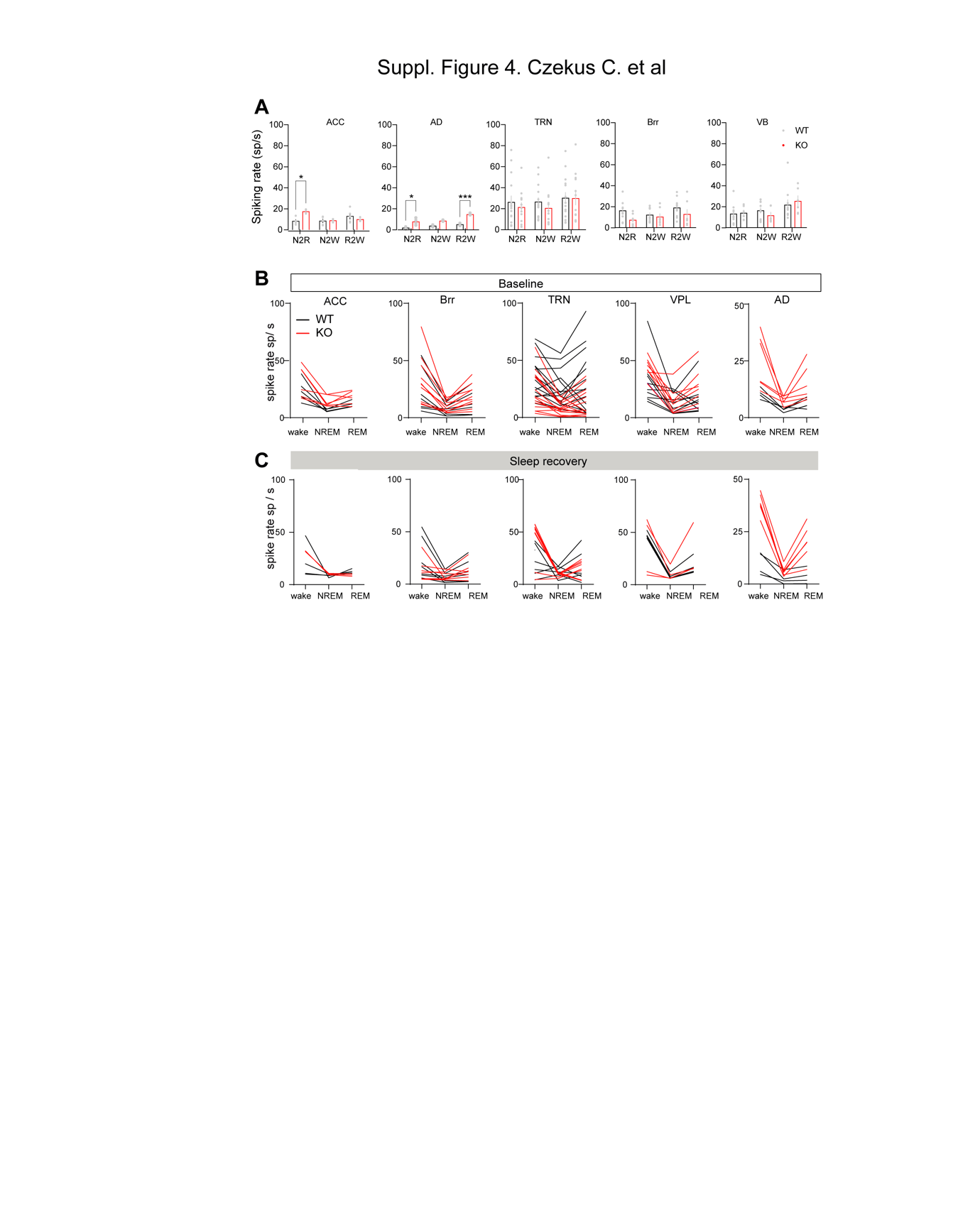


**Suppl. Figure 4. Spiking rate during the transition between different vigilance states.** **A.** Summary average data per animal collected from the anterior cingulate (ACC), the anterior dorsal thalamus (AD), the reticular thalamic nucleus (TRN), the sensory cortex (Brr) and the ventral posterolateral thalamic nucleus (VPL) in the period of 5 seconds before and after transition periods from NREM to REM sleep (N2R), NREM sleep to wakefulness (N2W) and from REM sleep to wakefulness (R2W). ACC: N2R *P= 0.016, n= 5 WT and 5 KO; AD: N2R *P= 0.018, R2W: ***P< 0.001, n= 2 and 4 for WT and KO); TRN (n=15 and 13 for WT and KO); Brr (n= 10, 7 for WT and KO); and VPL (n= 10, 6 for WT and KO) respectively. Significant levels were calculated using 2-way ANOVA and Bonferroni's multiple comparisons test. **B.** Summary data of individual isolated neurons across vigilance states in all recording sites of WT (black lines) and KO mouse (red lines) during baseline (BL) and **C.** during the first hour after sleep deprivation (SR, sleep recovery. Statistical differences were calculated using 2-way ANOVA and Bonferroni’s post hoc test to correct for multiple comparisons. Data represents the mean of individual values per animal +/- s.e.m.

| 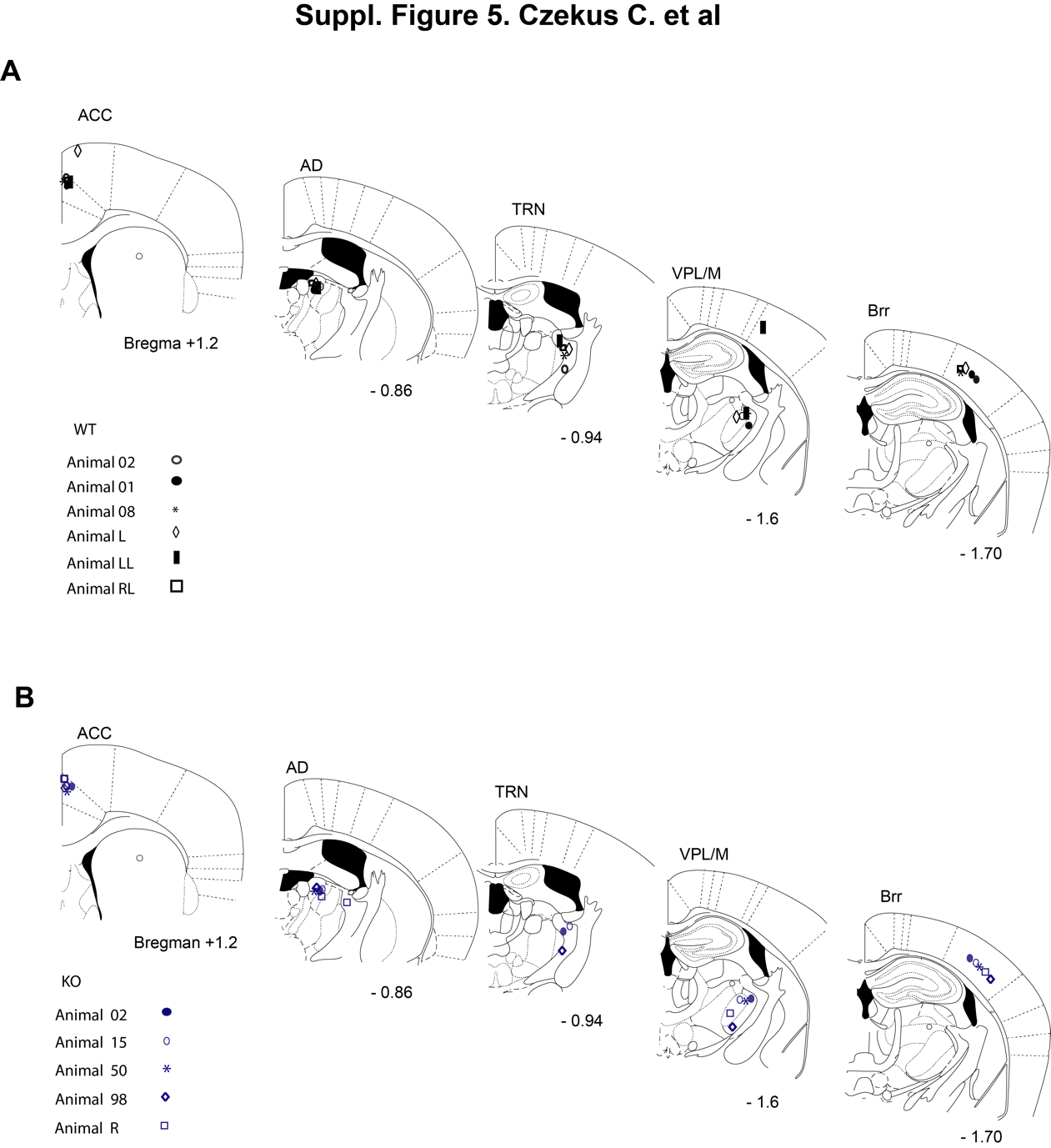 |
| --- |
| **Suppl. Figure 5. Electro placement in thalamocortical (TC) networks for multisite recordings in WT and KO treated with NAC. A.** Representative micrographs of cresyl violet staining showing the final tetrode locations in WT and KOs. **B.** in *Gclm* KO treated with NAC in the anterior cingulate (ACC), the anterior dorsal thalamus (AD), the reticular thalamic nucleus (TRN), the sensory cortex (Brr) and the ventral posterolateral thalamic nucleus (VPL). Different animals are designated with different signs. |
| \| **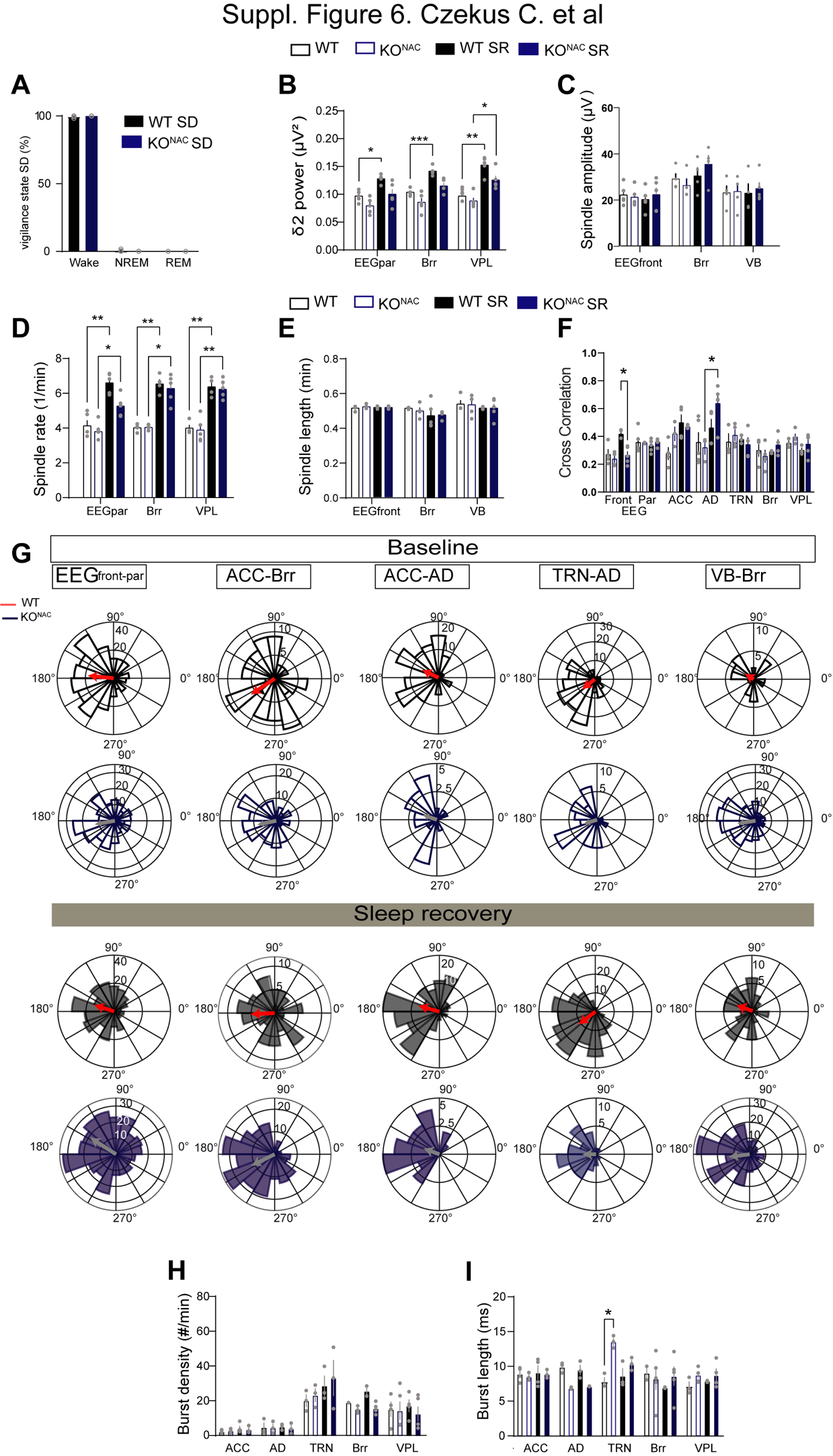** \| \| --- \| \| **Suppl. Figure 6. N-acetyl cysteine rescue of sleep and sleep spindles in *Gclm* KO mice. A.** Summary data of the averaged total quantities of Wake, NREM and REM sleep during the SD period (4 hs). n= 5 WT and 5KOs. **B.** Summary data of the normalized delta 2 (δ2) power collected during baseline (BL) and the sleep recovery period (SR) (F= 57.76 DFn= 3, DFd= 16) from: EEG^par^ [WT BL] - [WT SR] *P= 0.016, [WT SR] - [KO BL] **P= 0.006; Barrel (Brr) [WT BL] - [WT SR] ***P<0.001, [WT SR] - [KO BL] **P= 0.004, [WT SR] - [KO SR] *P= 0.3; VPL [WT BL] - [WT SR] **P= 0.00, [WT SR] - [KO BL] ***P , 0.001, [KO BL] - [KO SR] *P= 0.03; n=5 WT, 5 KO^NAC^. **C.** Spindle amplitude during NREM in baseline conditions (BL) and after sleep deprivation in the first hour of the sleep recovery period (SR). **D.** Spindle rate during BL and SR NREM sleep (F= 76.15 DFn= 3, DFd= 16) : EEG^par^ : [WT BL] - [WT SR] ** P= 0.001, [WT BL] -[KO SR] * P= 0.03, n= 5, 5 BL and n= 5, 5 SR; Brr = [WT BL] - [WT SR] **P= 0.005; [KO BL] -[KO SR] *P= 0.02, n= 4, 4 BL and n= 4, 5 SR; VPL [WT BL] - [WT SR] **P= 0.004; [KO BL] – [KO SR] *P= 0.003 n= 4, 4 BL and n= 5, 5. **E.** Spindle length during BL and SR NREM sleep. Note there were no differences in the values between WT and KO. **F**. Normalized cross-correlation between slow-waves and spindles during BL and SR in all recorded locations. EEG^front^ [WT SR] - [KO BL] **P= 0.0064, DF= 5.235; AD [KO BL] - [KO SR] *P= 0.031, DF= 6.98. **G.** Circular plots of the distribution of the phase coupling between SW phase (first pair) and the spindle amplitude on a distant cortical of thalamic nuclei (second pair) during the baseline (black line) and SR (blue line) of KO in all recorded locations. **H.** Burst density and **I**. burst length within the all recorded sites TRN [WT BL] - [KO BL] *P= 0.012, t= 7.12 DF= 3.87. *B-D* represent mean +/- s.e.m. from WT (black) and KO (bleu) mice in baseline (BL, open bars) and sleep recovery period (SR, filled bars). ACC (n= 4, 3); AD (n= 3, 3) TRN (n= 3, 3); Brr (n= 2, 5) and VPL (n= 3, 5) for WT and KO respectively. Significant differences calculated using two-way ANOVA and Bonferroni’s multiple comparison test. \|  \| **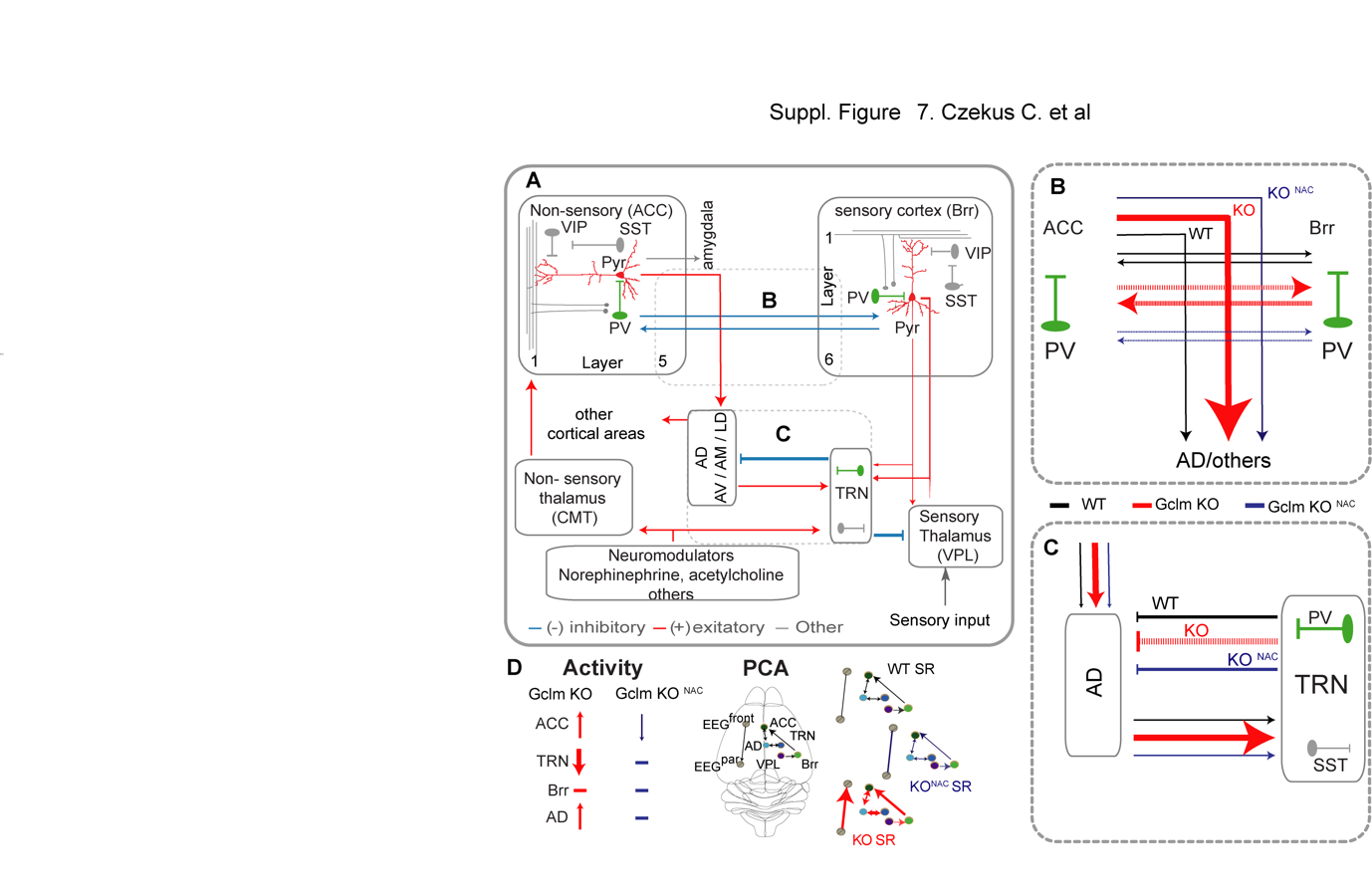** \| \| --- \| \| **Suppl. Figure 7.** **Schematic of parallel thalamocortical circuits and their connectivity in WT and KO mice.** **A.** Major connections between thalamocortical, cortico-cortical and subcortical circuits. In the cortex, Parvalbumin (PV), Somatostatin (SST) and Vasoactive Intestinal Peptide (VIP) expressing neurons inhibits the outputs of pyramidal neurons (Pyr) onto non-sensory (B) and sensory (C) thalamic neurons. Arrows are based on known anatomical connections. Non-sensory cortex, including the ACC, receives excitatory inputs (red lines) from the central medial thalamus (CMT), both of which receive excitatory and neuromodulatory inputs, including noradrenergic from the locus coeruleus (LC) and cholinergic from the pedunculopontine tegmentum (PPT) / laterodorsal tegmentum (LDT). Similarly, inputs from these areas modulate GABAergic cells (PV and SST) in the reticular thalamic nucleus (TRN) and its inhibitory tone (blue lines) onto non-sensory (anterior dorsal (AD), medial (AM), ventral (AV) and laterodorsal (LD) and sensory (ventroposterior lateral (VPL) and medial (VPM)) thalamic neurons. Sensory thalamus consequently modulates the activity of sensory neurons from the barrel cortex (Brr) that send feedback (excitatory) inputs to the TRN and the VPL/VPM to complete the sensory thalamocortical loop. See references for further details on these circuits^138-140^. **B.** Schematic representing changes in non-sensory corticothalamic neuron activities in wildtype and in *Gclm* KO without (red lines) and with antioxidative NAC treatment (KO^NAC^). **C.** Sensory thalamocortical model of TRN outputs/inputs to the non-sensory thalamic nuclei AD. Of note, no changes were observed in the connections to the sensory thalamus, likely due to the anterior localization of the TRN electrodes. **D.** Summary of the activity in the different recorded nuclei. Phase-frequency coupling (PAC) between interconnected areas were modulated by the phase of the SW (first listed location) and the amplitude of spindles (second listed location). Arrows illustrate the location of the neurons presumably driving the modulation (see Fig. 2E, 3C-D). Note that lack of full recovery in the EEG front-par and ACC-AD is likely due to the absence of delta modulation by the NAC treatment, suggesting that OxS has a greater effect on spindles recovery as compared to lower frequencies. \| |


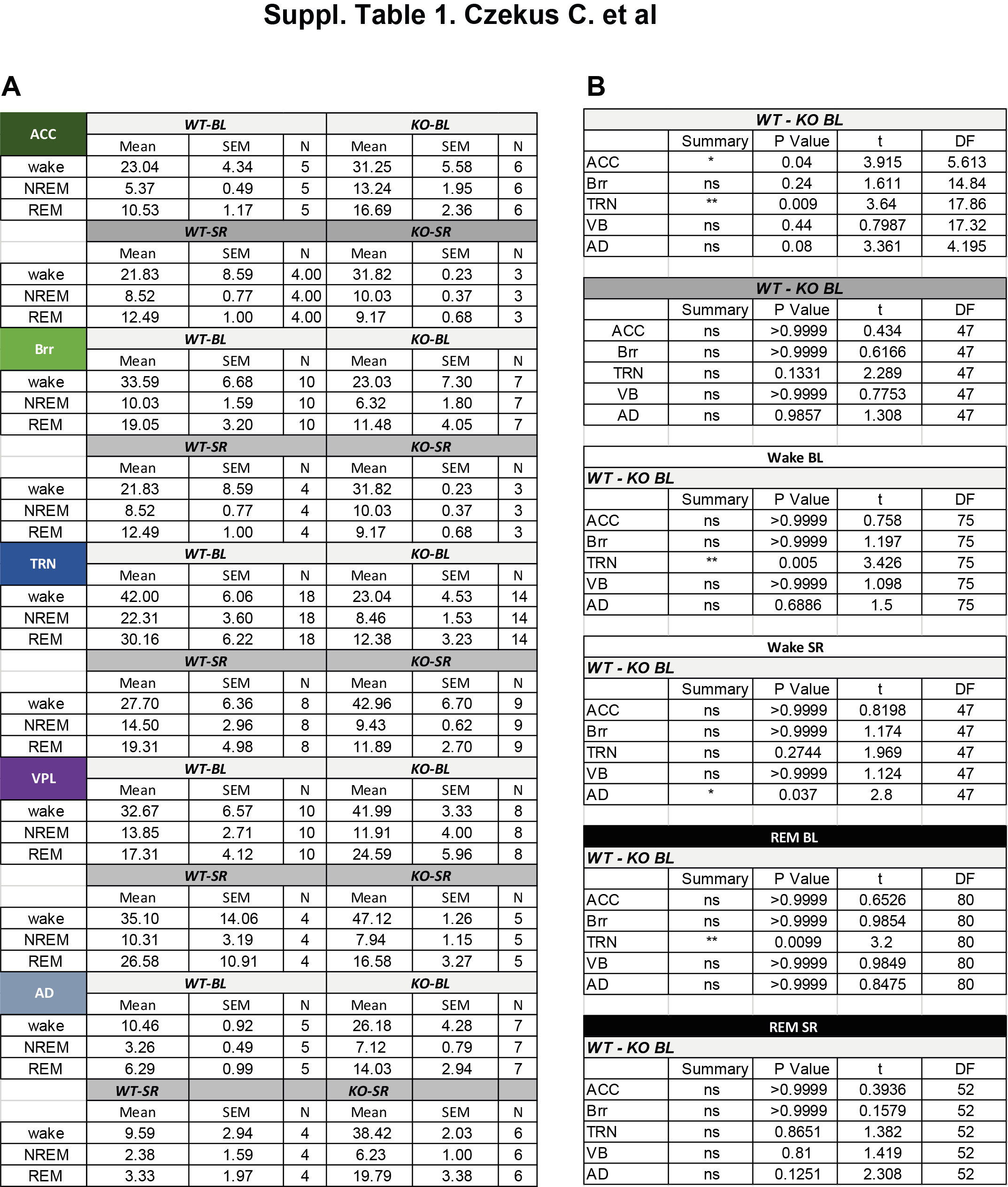


**Table1. Spiking rate activity across sleep wake cycle In *Gclm* WT and KO mice**. Data collected from spiking rate during all vigilance states: wake, NREM and REM sleep. Significance levels were calculated using 2-way ANOVA and Bonferroni's multiple comparisons test.
