# Supplementary figures and images for: Alterations in TRN-anterodorsal thalamocortical circuits affect sleep architecture and homeostatic processes in oxidative stress vulnerable Gclm−/− mice
Source: Mol Psychiatry. 2022 Jul 28;27(11):4394–406. doi: 10.1038/s41380-022-01700-w (PMC9734061; doi:10.1038/s41380-022-01700-w)

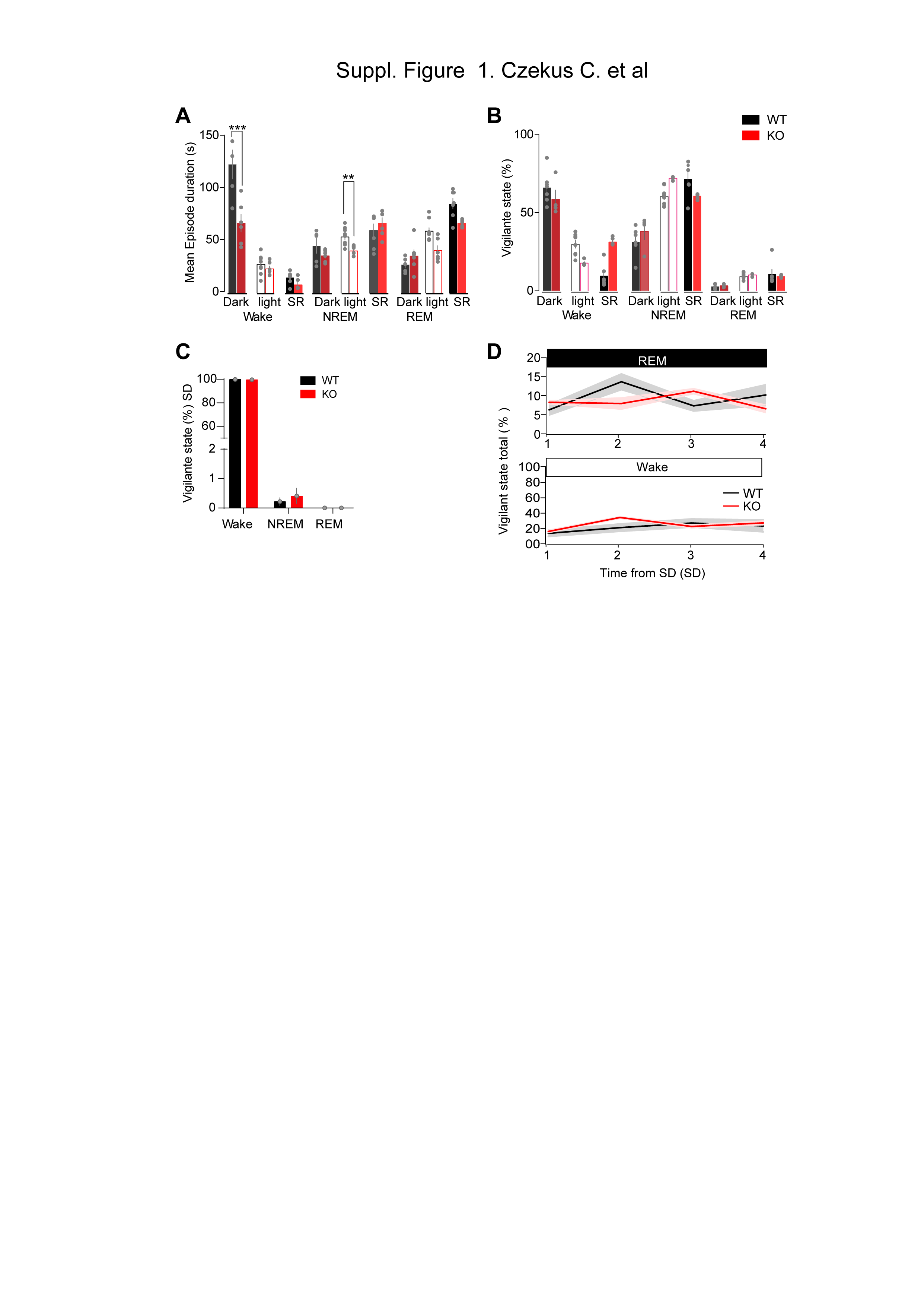

Supplement: Supplementary file 5 — Supplementary Figure 1 [file 41380_2022_1700_MOESM5_ESM.tif]

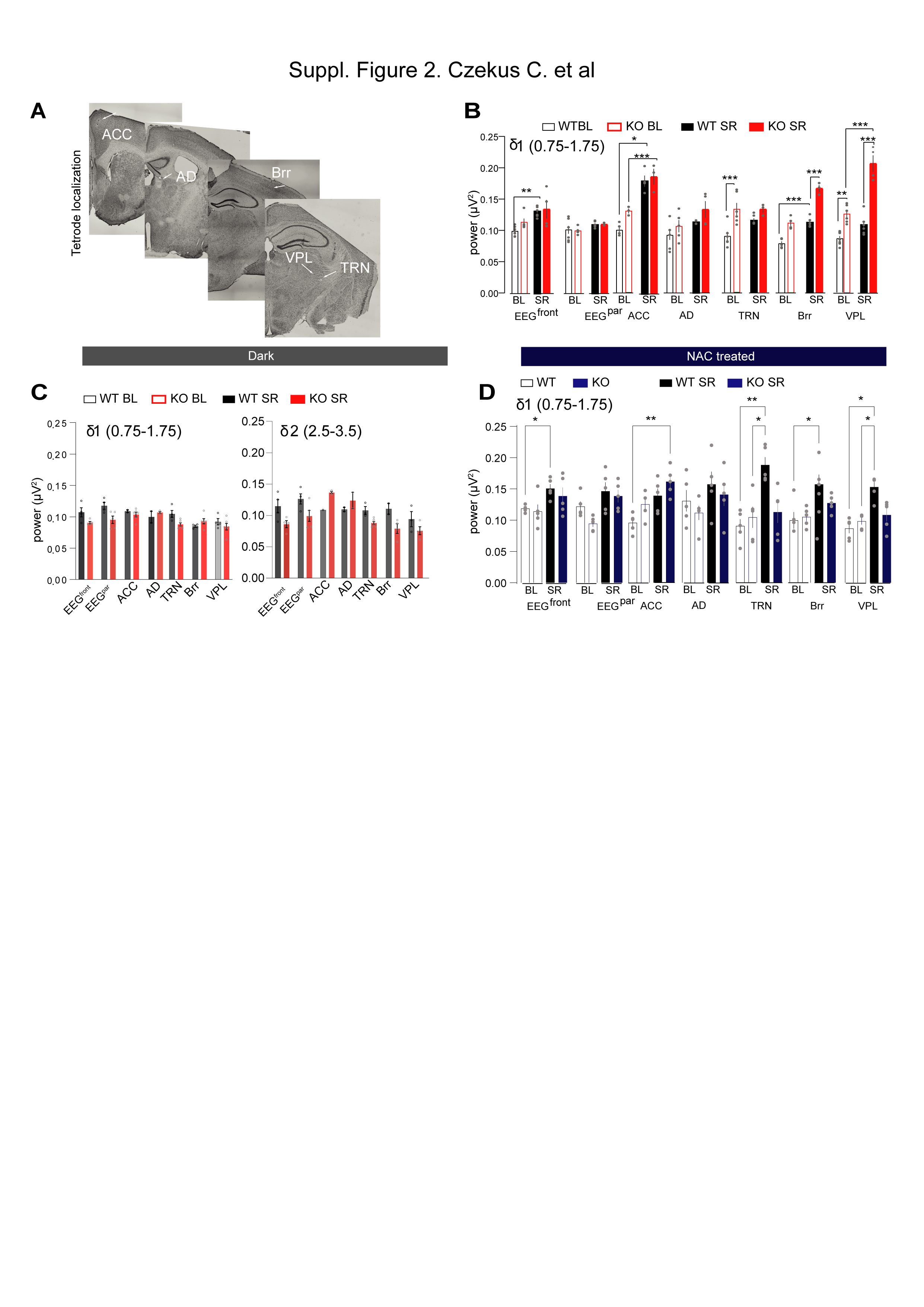

Supplement: Supplementary file 6 — Supplementary Figure 2 [file 41380_2022_1700_MOESM6_ESM.tif]

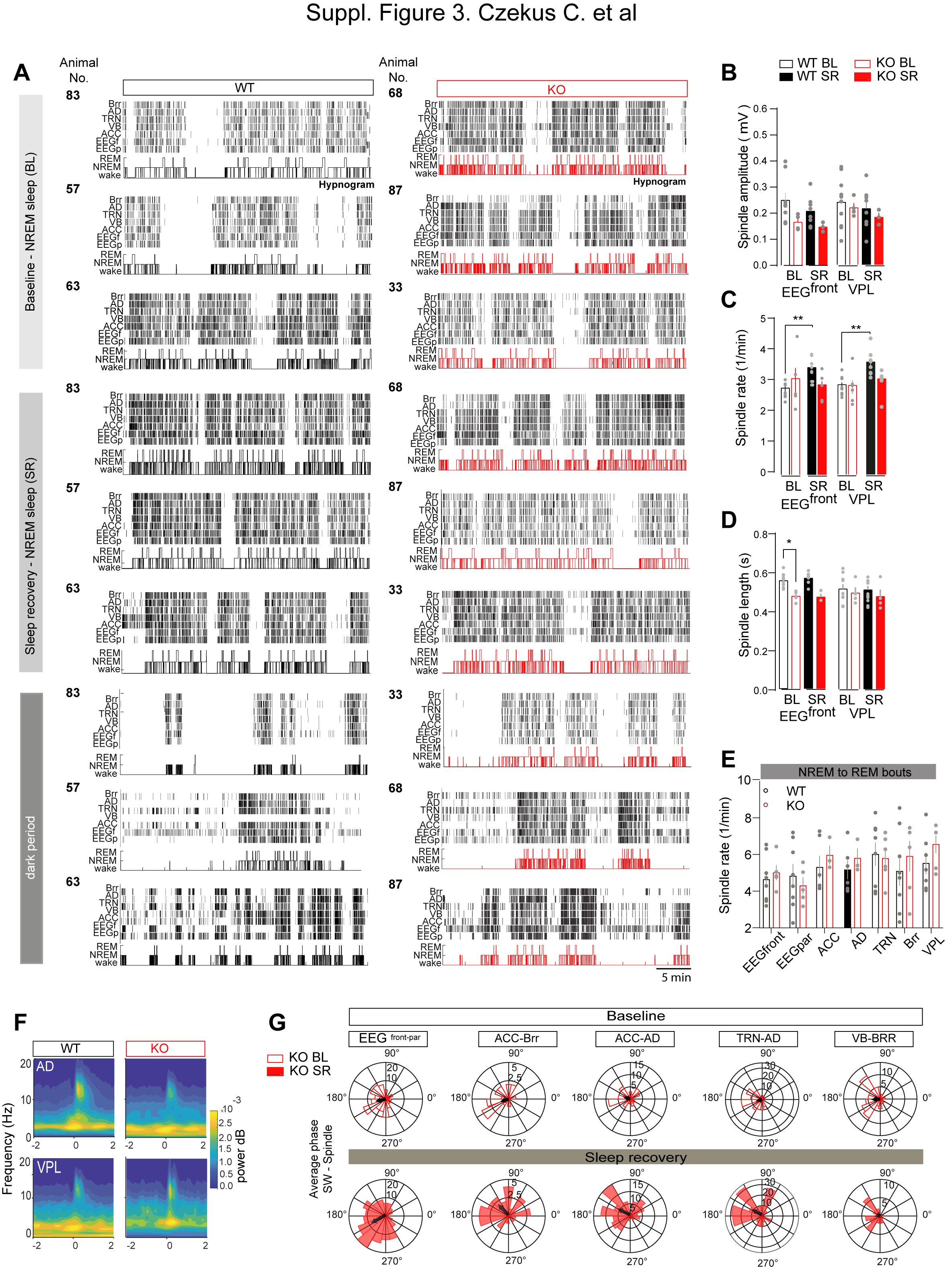

Supplement: Supplementary file 7 — Supplementary Figure 3 [file 41380_2022_1700_MOESM7_ESM.tif]

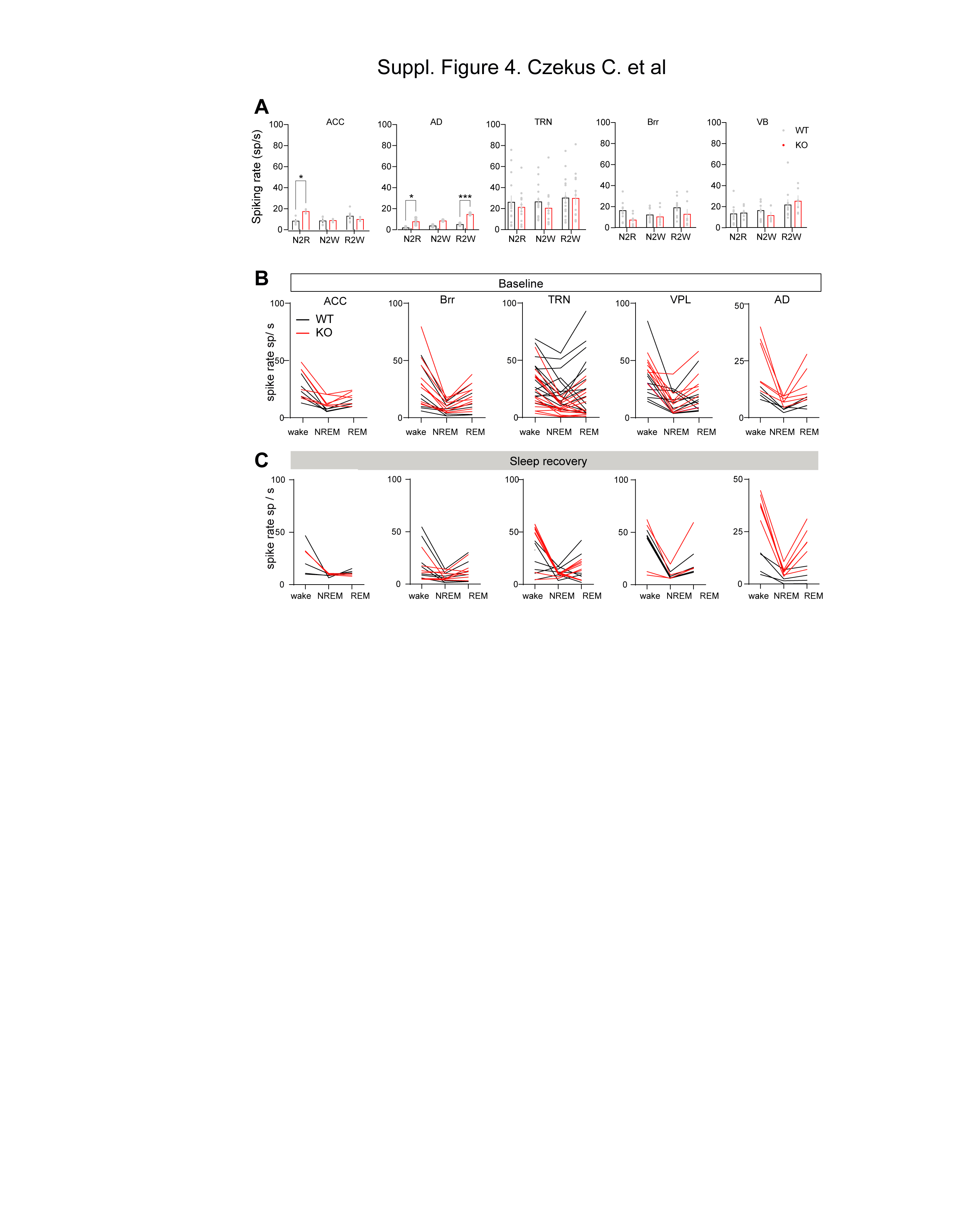

Supplement: Supplementary file 8 — Supplementary Figure 4 [file 41380_2022_1700_MOESM8_ESM.tif]

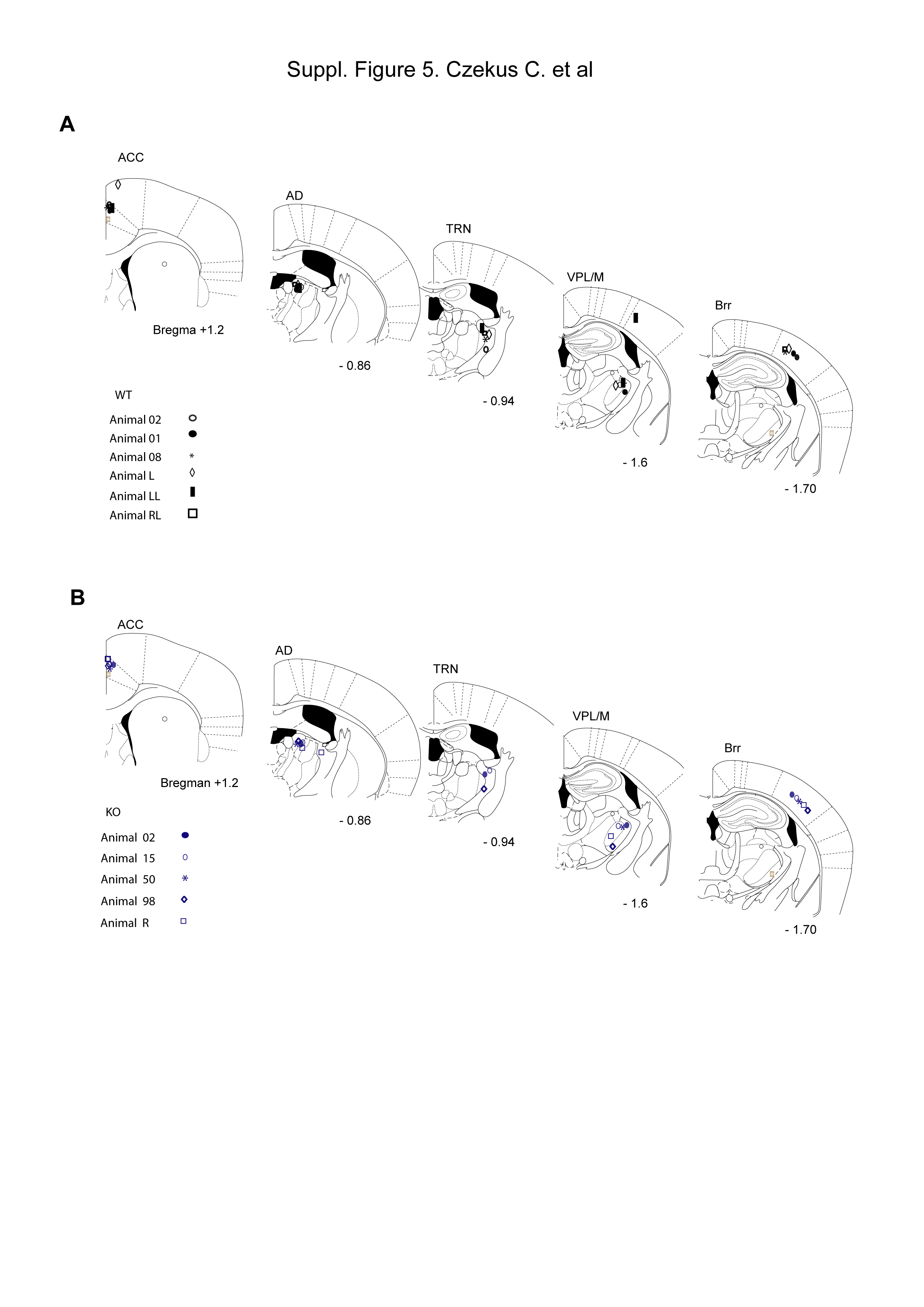

Supplement: Supplementary file 9 — Supplementary Figure 5 [file 41380_2022_1700_MOESM9_ESM.tif]

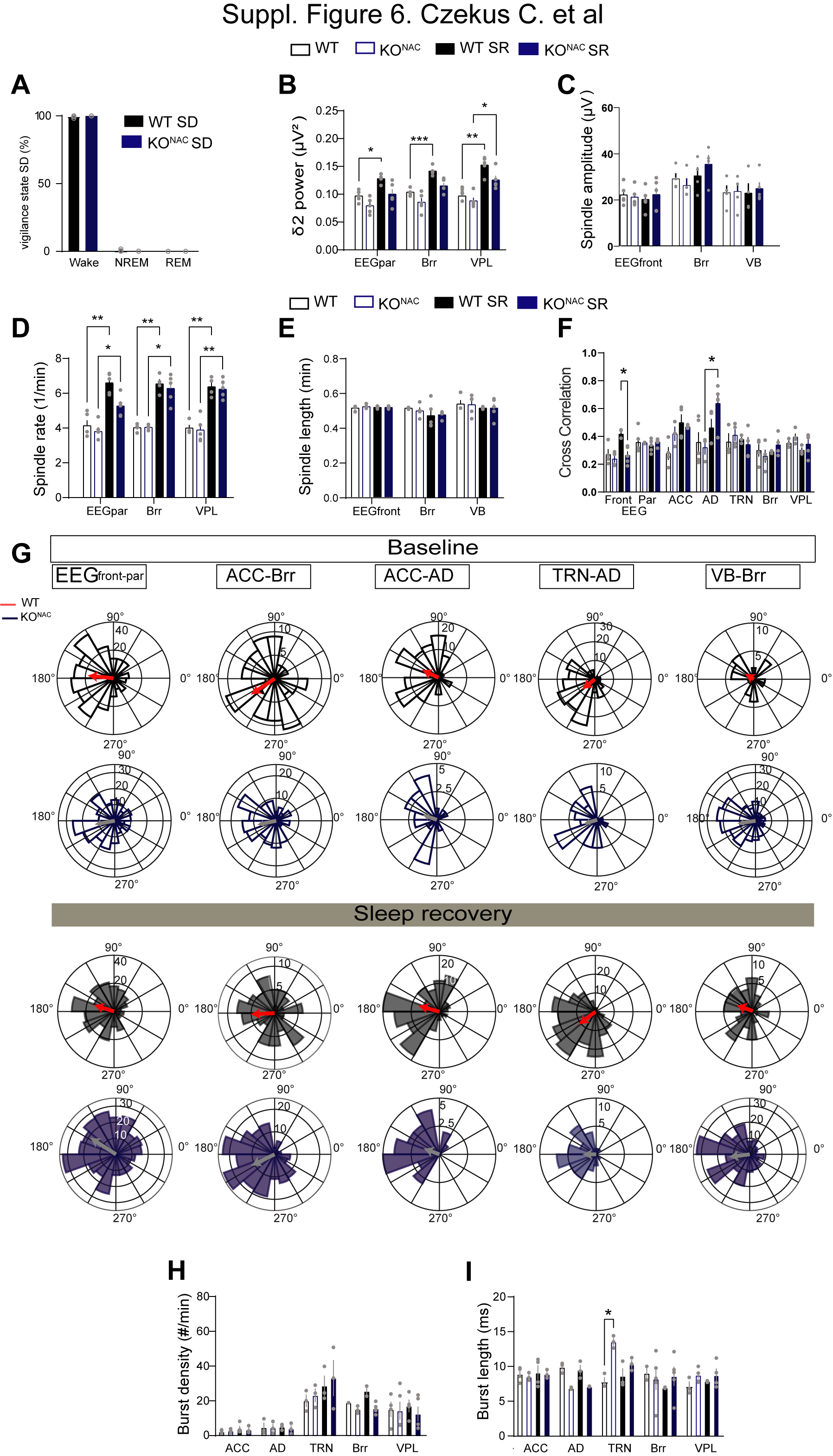

Supplement: Supplementary file 10 — Supplementary Figure 6 [file 41380_2022_1700_MOESM10_ESM.tif]

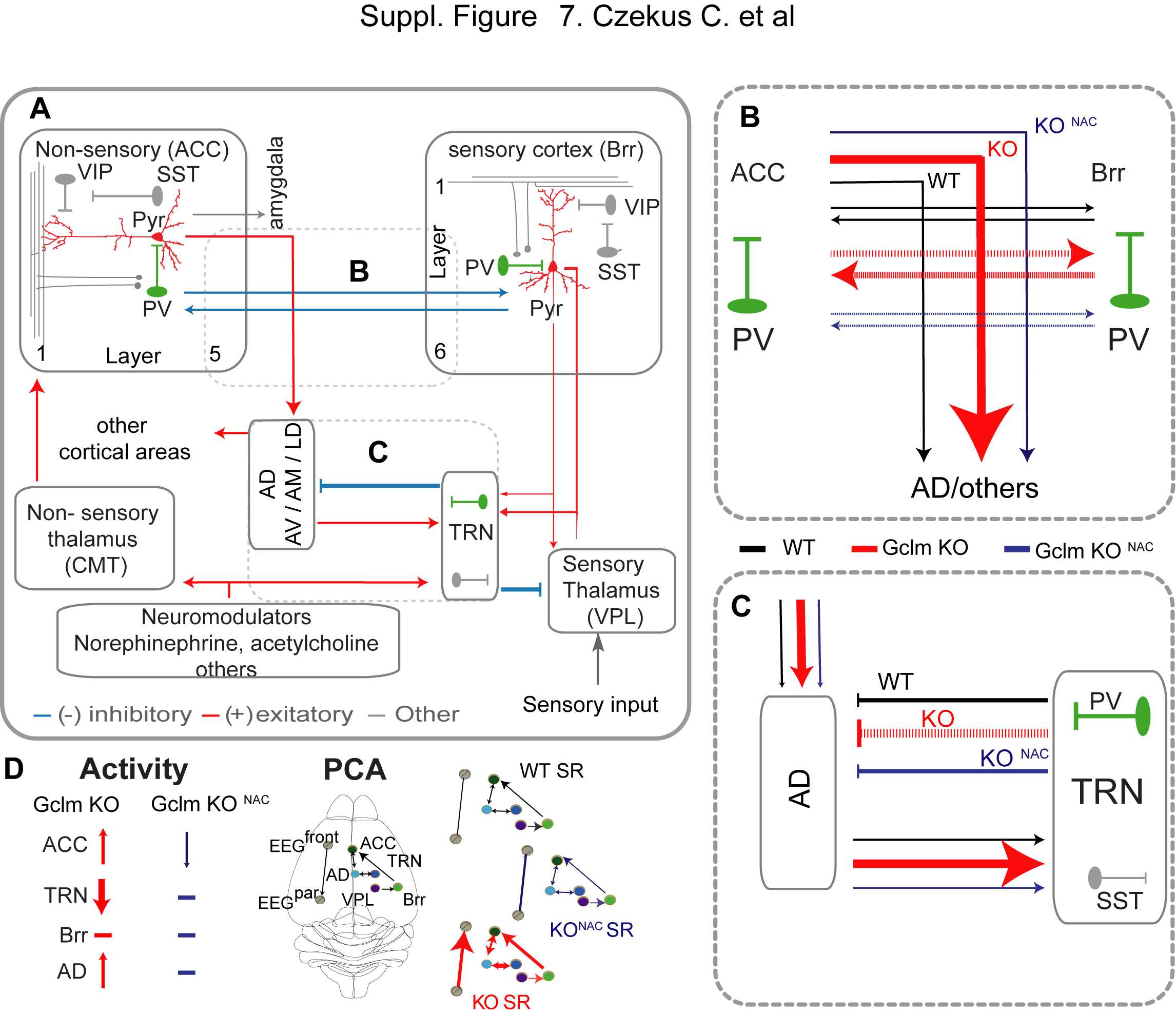

Supplement: Supplementary file 11 — Supplementary Figure 7 [file 41380_2022_1700_MOESM11_ESM.tif]
