# Supplementary material for: Alterations in TRN-anterodorsal thalamocortical circuits affect sleep architecture and homeostatic processes in oxidative stress vulnerable Gclm−/− mice
Source: Mol Psychiatry. 2022 Jul 28;27(11):4394–406. doi: 10.1038/s41380-022-01700-w (PMC9734061; doi:10.1038/s41380-022-01700-w)
Supplement: Supplementary file 12 — Supplementary Table 1 [file 41380_2022_1700_MOESM12_ESM.pdf]

Suppl. Table 1. Czekus C. et al.

**ACC**	***WT-BL***			***KO-BL***		
	Mean	SEM	N	Mean	SEM	N
wake	23.04	4.34	5	31.25	5.58	6
NREM	5.37	0.49	5	13.24	1.95	6
REM	10.53	1.17	5	16.69	2.36	6
	WT-SR			***KO-SR***		
	Mean	SEM	N	Mean	SEM	N
wake	21.83	8.59	4.00	31.82	0.23	3
NREM	8.52	0.77	4.00	10.03	0.37	3
REM	12.49	1.00	4.00	9.17	0.68	3
**Brr**	***WT-BL***			***KO-BL***		
	Mean	SEM	N	Mean	SEM	N
wake	33.59	6.68	10	23.03	7.30	7
NREM	10.03	1.59	10	6.32	1.80	7
REM	19.05	3.20	10	11.48	4.05	7
	WT-SR			***KO-SR***		
	Mean	SEM	N	Mean	SEM	N
wake	21.83	8.59	4	31.82	0.23	3
NREM	8.52	0.77	4	10.03	0.37	3
REM	12.49	1.00	4	9.17	0.68	3
**TRN**	***WT-BL***			***KO-BL***		
	Mean	SEM	N	Mean	SEM	N
wake	42.00	6.06	18	23.04	4.53	14
NREM	22.31	3.60	18	8.46	1.53	14
REM	30.16	6.22	18	12.38	3.23	14
	WT-SR			***KO-SR***		
	Mean	SEM	N	Mean	SEM	N
wake	27.70	6.36	8	42.96	6.70	9
NREM	14.50	2.96	8	9.43	0.62	9
REM	19.31	4.98	8	11.89	2.70	9
**VPL**	***WT-BL***			***KO-BL***		
	Mean	SEM	N	Mean	SEM	N
wake	32.67	6.57	10	41.99	3.33	8
NREM	13.85	2.71	10	11.91	4.00	8
REM	17.31	4.12	10	24.59	5.96	8
	WT-SR			***KO-SR***		
	Mean	SEM	N	Mean	SEM	N
wake	35.10	14.06	4	47.12	1.26	5
NREM	10.31	3.19	4	7.94	1.15	5
REM	26.58	10.91	4	16.58	3.27	5
**AD**	***WT-BL***			***KO-BL***		
	Mean	SEM	N	Mean	SEM	N
wake	10.46	0.92	5	26.18	4.28	7

NREM	3.26	0.49	5	7.12	0.79	7
REM	6.29	0.99	5	14.03	2.94	7
	WT-SR			***KO-SR***		
	Mean	SEM	N	Mean	SEM	N
wake	9.59	2.94	4	38.42	2.03	6
NREM	2.38	1.59	4	6.23	1.00	6
REM	3.33	1.97	4	19.79	3.38	6

WT - KO BL				
	Summary	P Value	t	DF
ACC	*	0.04	3.915	5.613
Brr	ns	0.24	1.611	14.84
TRN	**	0.009	3.64	17.86
VB	ns	0.44	0.7987	17.32
AD	ns	0.08	3.361	4.195

WT - KO BL				
	Summary	P Value	t	DF
ACC	ns	>0.9999	0.434	47
Brr	ns	>0.9999	0.6166	47
TRN	ns	0.1331	2.289	47
VB	ns	>0.9999	0.7753	47
AD	ns	0.9857	1.308	47

**Wake BL**				
WT - KO BL				
	Summary	P Value	t	DF
ACC	ns	>0.9999	0.758	75
Brr	ns	>0.9999	1.197	75
TRN	**	0.005	3.426	75
VB	ns	>0.9999	1.098	75
AD	ns	0.6886	1.5	75

**Wake SR**				
WT - KO BL				
	Summary	P Value	t	DF
ACC	ns	>0.9999	0.8198	47
Brr	ns	>0.9999	1.174	47
TRN	ns	0.2744	1.969	47
VB	ns	>0.9999	1.124	47
AD	*	0.037	2.8	47

REM BL

WT - KO BL				
	Summary	P Value	t	DF
ACC	ns	>0.9999	0.6526	80
Brr	ns	>0.9999	0.9854	80
TRN	**	0.0099	3.2	80
VB	ns	>0.9999	0.9849	80
AD	ns	>0.9999	0.8475	80

REM SR				
WT - KO BL				
	Summary	P Value	t	DF
ACC	ns	>0.9999	0.3936	52
Brr	ns	>0.9999	0.1579	52
TRN	ns	0.8651	1.382	52
VB	ns	0.81	1.419	52
AD	ns	0.1251	2.308	52
